# Identification and characterization of a silent mutation in RNA binding domain of N protein coding gene from SARS-CoV-2

**DOI:** 10.1186/s13104-020-05439-x

**Published:** 2021-01-06

**Authors:** Reza Zolfaghari Emameh, Mahyar Eftekhari, Hassan Nosrati, Jalal Heshmatnia, Reza Falak

**Affiliations:** 1 Department of Energy and Environmental Biotechnology, National Institute of Genetic Engineering and Biotechnology (NIGEB), 14965/161, Tehran, Iran; 2 Department of Clinical Medicine, Aarhus University Hospital, Aarhus, Denmark; 3 Chronic Respiratory Diseases Research Center (CRDRC), National Research Institute of Tuberculosis and Lung Diseases (NRITLD), Shahid Beheshti University of Medical Sciences, Tehran, Iran; 4 Immunology Research Center, Iran University of Medical Sciences, Tehran, Iran

**Keywords:** SARS-CoV-2, COVID-19, RNA binding domain, N protein, Mutation, Zinc, Thymine DNA glycosylase (TDG), Base excision repair

## Abstract

**Objective:**

This study describes the occurrence of a silent mutation in the RNA binding domain of nucleocapsid phosphoprotein (N protein) coding gene from SARS-CoV-2 that may consequence to a missense mutation by onset of another single nucleotide mutation.

**Results:**

In the DNA sequence isolated from severe acute respiratory syndrome (SARS-CoV-2) in Iran, a coding sequence for the RNA binding domain of N protein was detected. The comparison of Chinese and Iranian DNA sequences displayed that a thymine (T) was mutated to cytosine (C), so “TTG” from China was changed to “CTG” in Iran. Both DNA sequences from Iran and China have been encoded for leucine. In addition, the second T in “CTG” in the DNA or uracil (U) in “CUG” in the RNA sequences from Iran can be mutated to another C by a missense mutation resulting from thymine DNA glycosylase (TDG) of human and base excision repair mechanism to produce “CCG” encoding for proline, which consequently may increase the affinity of the RNA binding domain of N protein to viral RNA and improve the transcription rate, pathogenicity, evasion from human immunity system, spreading in the human body, and risk of human-to-human transmission rate of SARS-CoV-2.

## Introduction

Coronaviruses (CoVs) are the causative agent of diversity of infections in birds and mammals such as upper respiratory infection of chickens and enteritis in pigs and cows [1]. In addition to animals, human can be infected with different isolates of CoVs such as severe acute respiratory syndrome coronavirus (SARS-CoV) and Middle East respiratory syndrome coronavirus (MERS-CoV) with common cold, bronchitis, and pneumonia symptoms [2]. CoVs contain a positive-sense RNA strand with the genome size between 26.2 and 31.7 kb. The genomes of CoVs contain 3′ poly (A) and 5′ cap structures play as a mRNA in the translation activity of replicase enzyme [3]. It is specified that about 70% of the total size of the genome is occupied by replicase gene (20 kb) encoding for nonstructural proteins (nsp), while about 10 kb of the genome encodes for structural and accessory proteins. The studies have shown that the accessory proteins are not necessary for the viral replication, while are required in the CoVs pathogenesis and morphogenesis [4]. The CoVs contain four main structural proteins including spike (S), membrane (M), nucleocapsid (N), and envelope (E), which are expressed by the 3′ end of RNA genome [5]. S protein (150 kDa) contains a signal peptide sequence at the N-terminal with the ability to lead the S proteins to endoplasmic reticulum (ER) for glycosylation [6]. M protein (25–30 kDa) is the most abundant structural protein containing three transmembrane domains, which play a role in the virion formation. M proteins contain a small glycosylated N-terminal with the capability to bind to nucleocapsid so the M–N proteins binding may stabilize the complexes in the assembly of CoVs [7]. N protein (50–60 kDa) has the RNA binding capability by both N-terminal domain (NTD) and C-terminal domain (CTD) with two different mechanisms [8]. Three main factors have the crucial roles in the binding of N protein to RNA including phosphorylation of N protein, transcription regulatory signal (TRS), and genomic packaging signal (GPS). N protein binds to RNA, M protein, nsp3, replicase-transcriptase complex (RTC), which finally packages the RNA genome into CoVs. E protein (8–12 kDa) is a transmembrane protein with ion channel activity so that the lipid membrane charges affect the cation preference of these ion channels [9]. E protein facilitates the assemblage and release of virions from the infected cells. E protein is not necessary for CoV replication, while it is required for the CoV pathogenesis [10]. The studies revealed that SARS-CoV enters to the host cell using angiotensin converting enzyme 2 (ACE2) as the host receptor, while the host cell receptor for MERS-CoV is Dipeptidyl peptidase 4 (DPP4) [11].

Since December 2019, a novel member of CoVs have emerged from an outbreak in Wuhan, China, later on the causative agent was nominated as SARS-CoV-2 and the infection was nominated as coronavirus disease 2019 (COVID-19) [12]. Thereupon, the World Health Organization declared COVID-19 as a pandemic infection in March 2020 [13–15].

In this study and through a biodata mining approach, we evaluated a partial RNA binding domain of nucleocapsid protein (N protein) coding gene of SARS-CoV-2 isolated in Iran. In this coding gene, we found a silent thymine (T) to cytosine (C) mutation expressing leucine, which the encoded amino acid was similar to the query DNA sequence from Wuhan, China. This study alerts the incidence of second single nucleotide mutation in the subject coding sequence in Iran with the equal probability happened in the first T and changing middle T in DNA or U in RNA to C to form “CCG”. The encoded leucine will be changed to proline, which can influence the RNA binding activity of N protein and pathogenicity of SARS-CoV and induction of human immune system based on the 3D structure of newly emerged N protein.

## Main text

### Materials and methods

#### Sequence analysis

The coding gene for RNA binding domain of N protein of SARS-CoV-2 isolated in Iran was extracted from NCBI Virus database (https://www.ncbi.nlm.nih.gov/labs/virus/vssi/#/) [16]. The accession number of MT186676 was identified, which was related to a DNA sequence with 363 b in length, isolated in Iran, and annotated in the NCBI Virus database on 2020-03-13. The details of this annotated DNA sequence has been described as “Severe acute respiratory syndrome coronavirus 2 isolate SARS-CoV-2/1337/human/2020/IRN nucleocapsid phosphoprotein (N) gene, partial cds (Mokhtari Azad et al.)”. The accession number of encoded protein is QIK02784, which was nominated as partial nucleocapsid phosphoprotein with 122 amino acids in length.

The Iranian CDS sequence (MT186676) and the query CDS sequence from Wuhan, China (NC_045512.2) were aligned using Clustal Omega algorithm (https://www.ebi.ac.uk/Tools/msa/clustalo/) [17] of multiple sequence alignment (MSA). Then, the partial nucleocapsid phosphoprotein (QIK02784) from Iran and the query protein sequence from Wuhan, China (QIE07458) were aligned using Clustal Omega algorithm of MSA.

### Structural analysis

MT186676 related to the partial nucleocapsid phosphoprotein from Iran was analyzed in the RCSB Protein Data Bank (PDB) (https://www.rcsb.org/) [18]. In this data bank, a basic local alignment search tool (BLAST) was used to detect the most identical PDB identification number related to a crystalized protein to the Iranian protein sequence. In the next step, the NGL (WebGL) viewer [19] was employed to visualize NMR structure of the most identical protein structure to the partial nucleocapsid phosphoprotein from Iran.

## Results

### Sequence analysis

**Fig. 1:**
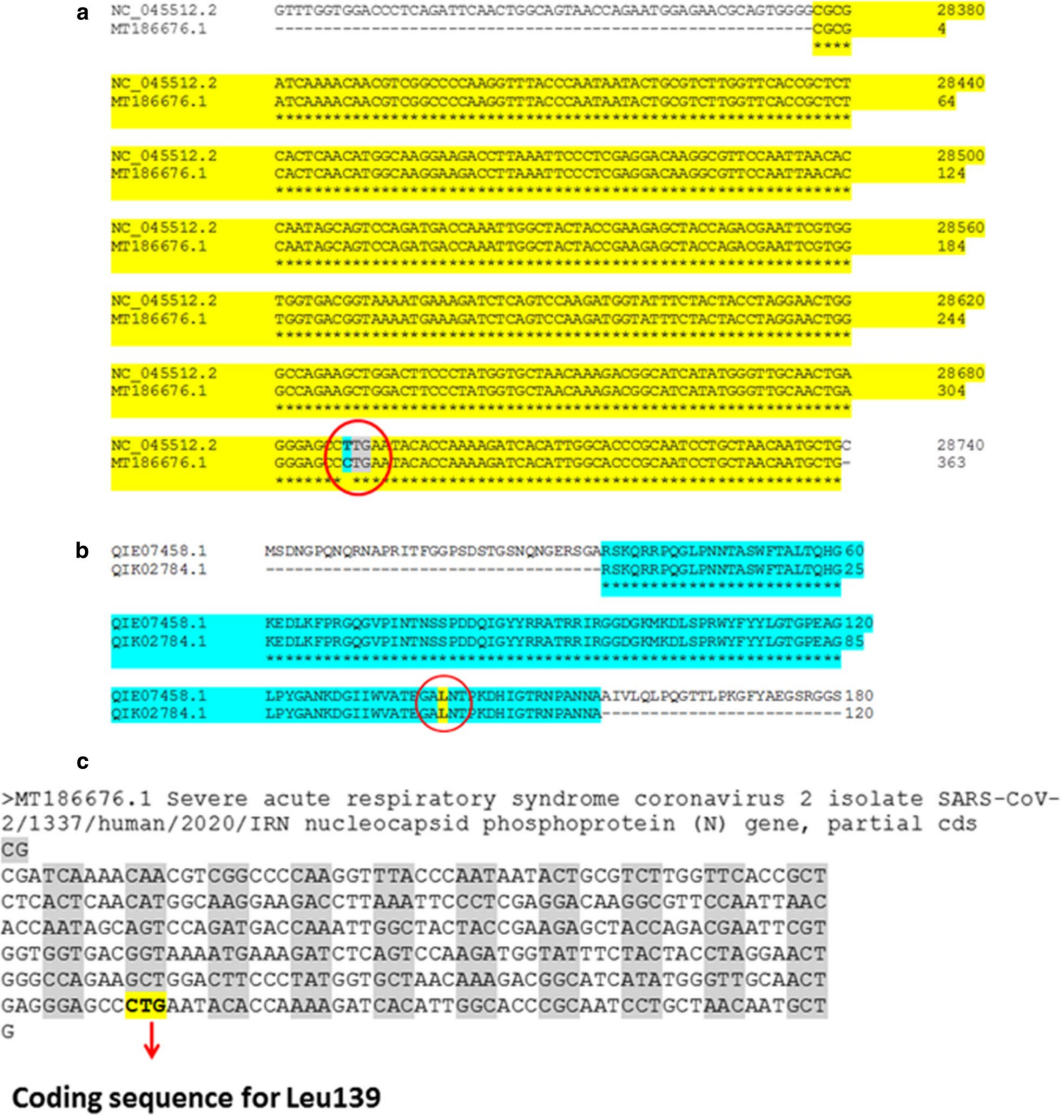
MSA analysis of RNA binding domain of N protein sequence. **a** MSA analysis of the CDS subject from Iran with the CDS query from Wuhan, China (yellow highlight); **b** MSA analysis of the partial RNA binding domain of N protein sequence from Iran with the query protein sequence from Wuhan, China (cyan highlight). Although both analyses show high similarity, a mismatch nucleotide is shown by the red circles in part A and B; and (**c**) The coding sequences of “TTG” in the CDS query sequence from Wuhan, China and “CTG” in the CDS subject sequence from Iran, which both encode for leucine

The MSA analysis of the CDS sequence coding for the partial RNA binding domain of N protein of SARS-CoV-2 isolated in Iran (MT186676) and the query CDS sequence from Wuhan, China (NC_045512.2) showed high similarity in both CDS sequences. A nucleotide mismatch was identified at nucleotide No. 312 that mutates T to C. The analysis revealed that both coding nucleotide sequences including “TTG” in the query CDS sequence from Wuhan, China and “CTG” in the subject CDS sequence from Iran encode for leucine (Fig. 1a). In addition, the MSA analysis of the query protein sequence from Wuhan, China (QIE07458) and the partial RNA binding domain of N protein from Iran (QIK02784) showed 100% similarity in both protein sequences (Fig. 1b and c) (Additional file 1: Fig. S1).

### Structural analysis

**Fig. 2:**
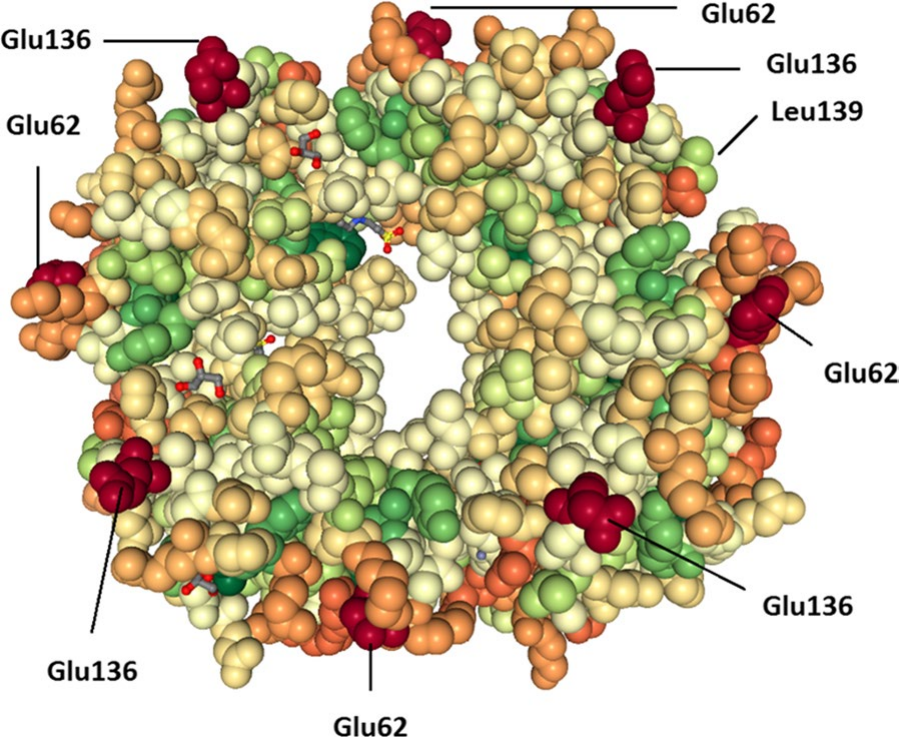
Protein structure of the RNA binding domain of N protein. The NMR structure entry ID: 6VYO was visualized by the NGL (WebGL) viewer. The exposure rate of eight glutamines including four Glu62 and four Glu136 in the tetrameric protein structure is higher than Leu139 encoded by the silent mutated sequence

**Fig. 3:**
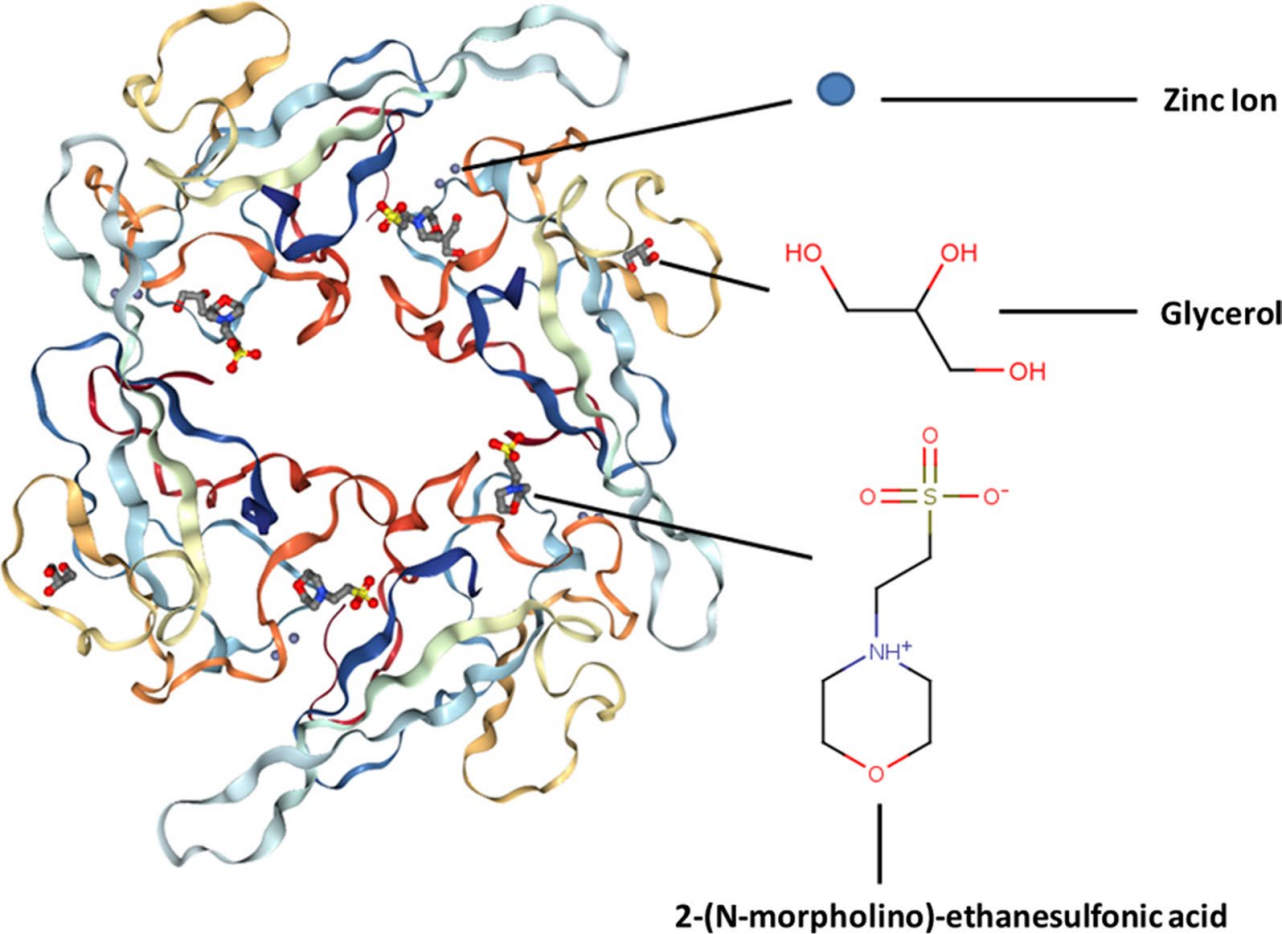
Tetrameric structure of the RNA binding domain of N protein from SARS-CoV-2. Each monomer contain one binding site for unique ligands including zinc ion (Zn^2+^), glycerol (GOL), and 2-(N-morpholino)-ethanesulfonic acid (MES)

The structural analysis of the partial RNA binding domain of N protein from Iran (QIK02784) in the RCSB PDB Protein Data Bank defined that the NMR entry ID: 6VYO belongs to the crystal structure of RNA binding domain of N protein from SARS-CoV-2 with *E*-value: 6.19076E−76, which in comparison to each other sequences showed 100% identity. This tetrameric protein structure contains four Glu62 and Glu136 with the highest exposure rate at the surface of the protein, while Leu139 encoded by the silent mutation (CTG) showed less exposure rate (Fig. 2). The analysis of the crystal structure of 6VYO revealed that the RNA binding domain of N protein from SARS-CoV-2 is a tetrameric structure, which each monomer contains three binding sites to unique ligands including zinc ion (Zn^2+^), glycerol (GOL), and 2-(N-morpholino)-ethanesulfonic acid (MES) (Fig. 3).

## Discussion

The comparison of both CDS and protein sequences for subject from Iran and query from Wuhan, China by the MSA method revealed that the subject sequence from Iran is the partial RNA binding domain of N protein from SARS-CoV-2. The studies revealed that N protein is phosphorylated, which triggers the binding affinity of N protein to the RNA genome of SARS-CoV-2 [5, 20]. In addition, both TRS and GPS are considered as the main factors of binding of N protein to the viral RNA. The further analysis revealed that the CDS subject sequence from Iran contains a silent mutation, which changed “TTG” in the CDS query sequence to “CTG”. Although both “TTG” and “CTG” encode for leucine, the occurrence of mutation in the middle T in “CTG” with the equal probability will change the encoded leucine (Leu139) to proline (Pro139).

In SARS-CoV-2, the mutations can occur under the pressure of the human immune system, which can be associated with increasing the transmission rate and pathogenicity of the virus. It is speculated that single mutation of first T in “TTG” to C to form “CTG” can be one of the changes occurred under the pressure of immune system. Changing the middle T to C to form “CCG” for encoding proline instead of leucine can be one of the evolutionary strategies of SARS-CoV-2. It is speculated that the middle T in “CTG” or middle U in “CUG” have undergo a G:T (in DNA) or G:U (in RNA) mismatch, which was repaired by employing thymine DNA glycosylase (TDG) of human and base excision repair system with replacing the middle T to C. Although TDG is a crucial enzyme in the human DNA repair system, it seems that TDG can be employed within the SARS-CoV-2 genome organization during the COVID-19 infection in human as well. Further biochemical analysis of constituent amino acids of the RNA binding domain of N protein from SARS-CoV-2 defined that not only the exposure rate of the RNA binding domain of N protein is increased with replacement of leucine with proline, but also increase the chance of immune evasion of this protein [21]. Therefore, this missense mutation may increase the binding rate of N protein to the viral RNA; improving the transcription rate of the viral RNA, escaping from the human immune system, and consequently raise incidence of COVID-19 cases with higher pathogenicity rate in Iran.

The probability of emerge of next generation of SARS-CoV-2 in Iran through this missense mutation has the potential to be transmitted worldwide by human-to-human contacts as well. This happening may prepare more human-to-human transmission cases, which consequently rise the risk of evolution of SARS-CoV-2 by accumulation of silent mutations leading to a missense mutation [22]. The study of binding sites of unique ligands including Zn^2+^, GOL, and MES to the RNA binding domain of N protein demonstrated Zn^2+^ coordination has a major role in the geometry of tetrahedral structure of protein; GOL plays the role in the stability of protein, and MES is associated with the preparation of buffering condition for the protein [23]. Therefore, the ligand binding information can provide the invaluable opportunities to chelate Zn^2+^ by appropriate inhibitors as an antiviral therapy. The chelation of Zn^2+^ and the early stage inhibition of replication had been studied in dengue virus [24]. On the other hand, another study showed that RNA-dependent RNA polymerase (RdRp) of nidovirus, a CoV member, is inhibited by Zn^2+^ [25]. Hence, focusing on Zn^2+^, Zn^2+^ chelating agents, and inhibition of Zn^2+^-associated enzymes and proteins [24–26] potentially may be a pharmaceutical measure to treat COVID-19.

## Conclusion

In conclusion, the more opportunities for human-to-human transmission,the more occurrence of missense mutation risk, improving the replication and transcription rates, and raising the virulence and pathogenicity of SARS-CoV-2. In addition, Zn^2+^ and Zn^2+^ inhibition studies have the potentials to become a medication compound and strategy against COVID-19. Moreover, our results revealed that TDG of human can be employed by SARS-CoV-2 during the genome organization and repairing leading to emerge of another mutated SARS-CoV-2 strain, which this mechanism has not reported yet by molecular virology studies of SARS-CoV-2 and needs to be examined by further experimental studies in the future.

## Limitations

This study was performed based on the bioinformatics and computational analyses. In addition, further mutations may be occurred in the genome of SARS-CoV-2 to increase the transcription rate of viral RNA, pathogenicity of virus, evasion from the human immune system, spreading in the human body, binding of virus to cell receptors, and human-to-human transmission rate.

## Supplementary Information

**Additional file 1:** **Fig. S1 **(A) MSA between the CDS coding for the RNA binding domain of N protein from Wuhan, China, and the CDS coding for the RNA binding domain of N protein from Iran. (B) MSA between the protein sequence of the RNA binding domain of N protein from Wuhan, China, and the protein sequence of the RNA binding domain of N protein from Iran.

## Data Availability

All data analyzed in this study were prepared from databases including NCBI Virus and RCSB PDB Protein Data Bank were included in this article. These IDs are including MT186676, QIK02784, NC_045512.2, and QIE07458 from NCBI database and 6VYO from RCSB PDB Protein Data Bank.

## Abbreviations

ACE2: Angiotensin converting enzyme 2
BLAST: Basic Local Alignment Search Tool
C: Cytosine
CDS: Coding sequence
COVID-19: Coronavirus disease 2019
DPP4: Dipeptidyl peptidase 4
E: Envelope
ER: Endoplasmic reticulum
Glu: Glutamine
GOL: Glycerol
Leu: Leucine
M: Membrane
MERS-CoV: Middle Eastern Respiratory Syndrome Coronavirus
MES: 2-(N-morpholino)-ethanesulfonic acid
MSA: Multiple sequence alignment
N: Nucleocapsid
NCBI: National Center for Biotechnology Information
NMR: Nuclear magnetic resonance
nsp: Nonstructural protein
ORF: Open reading frame
PDB: Protein data bank
Pro: Proline
RdRP: RNA-dependent RNA polymerase
RNA: Ribonucleic acid
RTC: Replication-transcription complexes
S: Spike
SARS-CoV-2: Severe Acute Respiratory Syndrome Coronavirus 2
T: Thymine
TDG: Thymine DNA glycosylase
U: Uracil
Zn^2^^+^: Zinc ion
