# Supplementary material for: Identification and characterization of a silent mutation in RNA binding domain of N protein coding gene from SARS-CoV-2
Source: BMC Res Notes. 2021 Jan 6;14:10. doi: 10.1186/s13104-020-05439-x (PMC7787625; doi:10.1186/s13104-020-05439-x)
Supplement: Supplementary file 1 — Additional file 1: Fig. S1 (A) MSA between the CDS coding for the RNA binding domain of N protein from Wuhan, China, and the CDS coding for the RNA binding domain of N protein from Iran. (B) MSA between the protein sequence of the RNA binding domain of N protein from Wuhan, China, and the protein sequence of the RNA binding domain of N protein from Iran. [file 13104_2020_5439_MOESM1_ESM.docx]

**Fig. S1 (A) MSA between the CDS coding for the RNA binding domain of N protein from Wuhan, China, and the CDS coding for the RNA binding domain of N protein from Iran.**

NC_045512.2 ATTAAAGGTTTATACCTTCCCAGGTAACAAACCAACCAACTTTCGATCTCTTGTAGATCT 60

MT186676.1 ------------------------------------------------------------ 0

NC_045512.2 GTTCTCTAAACGAACTTTAAAATCTGTGTGGCTGTCACTCGGCTGCATGCTTAGTGCACT 120

MT186676.1 ------------------------------------------------------------ 0

NC_045512.2 CACGCAGTATAATTAATAACTAATTACTGTCGTTGACAGGACACGAGTAACTCGTCTATC 180

MT186676.1 ------------------------------------------------------------ 0

NC_045512.2 TTCTGCAGGCTGCTTACGGTTTCGTCCGTGTTGCAGCCGATCATCAGCACATCTAGGTTT 240

MT186676.1 ------------------------------------------------------------ 0

NC_045512.2 CGTCCGGGTGTGACCGAAAGGTAAGATGGAGAGCCTTGTCCCTGGTTTCAACGAGAAAAC 300

MT186676.1 ------------------------------------------------------------ 0

NC_045512.2 ACACGTCCAACTCAGTTTGCCTGTTTTACAGGTTCGCGACGTGCTCGTACGTGGCTTTGG 360

MT186676.1 ------------------------------------------------------------ 0

NC_045512.2 AGACTCCGTGGAGGAGGTCTTATCAGAGGCACGTCAACATCTTAAAGATGGCACTTGTGG 420

MT186676.1 ------------------------------------------------------------ 0

NC_045512.2 CTTAGTAGAAGTTGAAAAAGGCGTTTTGCCTCAACTTGAACAGCCCTATGTGTTCATCAA 480

MT186676.1 ------------------------------------------------------------ 0

NC_045512.2 ACGTTCGGATGCTCGAACTGCACCTCATGGTCATGTTATGGTTGAGCTGGTAGCAGAACT 540

MT186676.1 ------------------------------------------------------------ 0

NC_045512.2 CGAAGGCATTCAGTACGGTCGTAGTGGTGAGACACTTGGTGTCCTTGTCCCTCATGTGGG 600

MT186676.1 ------------------------------------------------------------ 0

NC_045512.2 CGAAATACCAGTGGCTTACCGCAAGGTTCTTCTTCGTAAGAACGGTAATAAAGGAGCTGG 660

MT186676.1 ------------------------------------------------------------ 0

NC_045512.2 TGGCCATAGTTACGGCGCCGATCTAAAGTCATTTGACTTAGGCGACGAGCTTGGCACTGA 720

MT186676.1 ------------------------------------------------------------ 0

NC_045512.2 TCCTTATGAAGATTTTCAAGAAAACTGGAACACTAAACATAGCAGTGGTGTTACCCGTGA 780

MT186676.1 ------------------------------------------------------------ 0

NC_045512.2 ACTCATGCGTGAGCTTAACGGAGGGGCATACACTCGCTATGTCGATAACAACTTCTGTGG 840

MT186676.1 ------------------------------------------------------------ 0

NC_045512.2 CCCTGATGGCTACCCTCTTGAGTGCATTAAAGACCTTCTAGCACGTGCTGGTAAAGCTTC 900

MT186676.1 ------------------------------------------------------------ 0

NC_045512.2 ATGCACTTTGTCCGAACAACTGGACTTTATTGACACTAAGAGGGGTGTATACTGCTGCCG 960

MT186676.1 ------------------------------------------------------------ 0

NC_045512.2 TGAACATGAGCATGAAATTGCTTGGTACACGGAACGTTCTGAAAAGAGCTATGAATTGCA 1020

MT186676.1 ------------------------------------------------------------ 0

NC_045512.2 GACACCTTTTGAAATTAAATTGGCAAAGAAATTTGACACCTTCAATGGGGAATGTCCAAA 1080

MT186676.1 ------------------------------------------------------------ 0

NC_045512.2 TTTTGTATTTCCCTTAAATTCCATAATCAAGACTATTCAACCAAGGGTTGAAAAGAAAAA 1140

MT186676.1 ------------------------------------------------------------ 0

NC_045512.2 GCTTGATGGCTTTATGGGTAGAATTCGATCTGTCTATCCAGTTGCGTCACCAAATGAATG 1200

MT186676.1 ------------------------------------------------------------ 0

NC_045512.2 CAACCAAATGTGCCTTTCAACTCTCATGAAGTGTGATCATTGTGGTGAAACTTCATGGCA 1260

MT186676.1 ------------------------------------------------------------ 0

NC_045512.2 GACGGGCGATTTTGTTAAAGCCACTTGCGAATTTTGTGGCACTGAGAATTTGACTAAAGA 1320

MT186676.1 ------------------------------------------------------------ 0

NC_045512.2 AGGTGCCACTACTTGTGGTTACTTACCCCAAAATGCTGTTGTTAAAATTTATTGTCCAGC 1380

MT186676.1 ------------------------------------------------------------ 0

NC_045512.2 ATGTCACAATTCAGAAGTAGGACCTGAGCATAGTCTTGCCGAATACCATAATGAATCTGG 1440

MT186676.1 ------------------------------------------------------------ 0

NC_045512.2 CTTGAAAACCATTCTTCGTAAGGGTGGTCGCACTATTGCCTTTGGAGGCTGTGTGTTCTC 1500

MT186676.1 ------------------------------------------------------------ 0

NC_045512.2 TTATGTTGGTTGCCATAACAAGTGTGCCTATTGGGTTCCACGTGCTAGCGCTAACATAGG 1560

MT186676.1 ------------------------------------------------------------ 0

NC_045512.2 TTGTAACCATACAGGTGTTGTTGGAGAAGGTTCCGAAGGTCTTAATGACAACCTTCTTGA 1620

MT186676.1 ------------------------------------------------------------ 0

NC_045512.2 AATACTCCAAAAAGAGAAAGTCAACATCAATATTGTTGGTGACTTTAAACTTAATGAAGA 1680

MT186676.1 ------------------------------------------------------------ 0

NC_045512.2 GATCGCCATTATTTTGGCATCTTTTTCTGCTTCCACAAGTGCTTTTGTGGAAACTGTGAA 1740

MT186676.1 ------------------------------------------------------------ 0

NC_045512.2 AGGTTTGGATTATAAAGCATTCAAACAAATTGTTGAATCCTGTGGTAATTTTAAAGTTAC 1800

MT186676.1 ------------------------------------------------------------ 0

NC_045512.2 AAAAGGAAAAGCTAAAAAAGGTGCCTGGAATATTGGTGAACAGAAATCAATACTGAGTCC 1860

MT186676.1 ------------------------------------------------------------ 0

NC_045512.2 TCTTTATGCATTTGCATCAGAGGCTGCTCGTGTTGTACGATCAATTTTCTCCCGCACTCT 1920

MT186676.1 ------------------------------------------------------------ 0

NC_045512.2 TGAAACTGCTCAAAATTCTGTGCGTGTTTTACAGAAGGCCGCTATAACAATACTAGATGG 1980

MT186676.1 ------------------------------------------------------------ 0

NC_045512.2 AATTTCACAGTATTCACTGAGACTCATTGATGCTATGATGTTCACATCTGATTTGGCTAC 2040

MT186676.1 ------------------------------------------------------------ 0

NC_045512.2 TAACAATCTAGTTGTAATGGCCTACATTACAGGTGGTGTTGTTCAGTTGACTTCGCAGTG 2100

MT186676.1 ------------------------------------------------------------ 0

NC_045512.2 GCTAACTAACATCTTTGGCACTGTTTATGAAAAACTCAAACCCGTCCTTGATTGGCTTGA 2160

MT186676.1 ------------------------------------------------------------ 0

NC_045512.2 AGAGAAGTTTAAGGAAGGTGTAGAGTTTCTTAGAGACGGTTGGGAAATTGTTAAATTTAT 2220

MT186676.1 ------------------------------------------------------------ 0

NC_045512.2 CTCAACCTGTGCTTGTGAAATTGTCGGTGGACAAATTGTCACCTGTGCAAAGGAAATTAA 2280

MT186676.1 ------------------------------------------------------------ 0

NC_045512.2 GGAGAGTGTTCAGACATTCTTTAAGCTTGTAAATAAATTTTTGGCTTTGTGTGCTGACTC 2340

MT186676.1 ------------------------------------------------------------ 0

NC_045512.2 TATCATTATTGGTGGAGCTAAACTTAAAGCCTTGAATTTAGGTGAAACATTTGTCACGCA 2400

MT186676.1 ------------------------------------------------------------ 0

NC_045512.2 CTCAAAGGGATTGTACAGAAAGTGTGTTAAATCCAGAGAAGAAACTGGCCTACTCATGCC 2460

MT186676.1 ------------------------------------------------------------ 0

NC_045512.2 TCTAAAAGCCCCAAAAGAAATTATCTTCTTAGAGGGAGAAACACTTCCCACAGAAGTGTT 2520

MT186676.1 ------------------------------------------------------------ 0

NC_045512.2 AACAGAGGAAGTTGTCTTGAAAACTGGTGATTTACAACCATTAGAACAACCTACTAGTGA 2580

MT186676.1 ------------------------------------------------------------ 0

NC_045512.2 AGCTGTTGAAGCTCCATTGGTTGGTACACCAGTTTGTATTAACGGGCTTATGTTGCTCGA 2640

MT186676.1 ------------------------------------------------------------ 0

NC_045512.2 AATCAAAGACACAGAAAAGTACTGTGCCCTTGCACCTAATATGATGGTAACAAACAATAC 2700

MT186676.1 ------------------------------------------------------------ 0

NC_045512.2 CTTCACACTCAAAGGCGGTGCACCAACAAAGGTTACTTTTGGTGATGACACTGTGATAGA 2760

MT186676.1 ------------------------------------------------------------ 0

NC_045512.2 AGTGCAAGGTTACAAGAGTGTGAATATCACTTTTGAACTTGATGAAAGGATTGATAAAGT 2820

MT186676.1 ------------------------------------------------------------ 0

NC_045512.2 ACTTAATGAGAAGTGCTCTGCCTATACAGTTGAACTCGGTACAGAAGTAAATGAGTTCGC 2880

MT186676.1 ------------------------------------------------------------ 0

NC_045512.2 CTGTGTTGTGGCAGATGCTGTCATAAAAACTTTGCAACCAGTATCTGAATTACTTACACC 2940

MT186676.1 ------------------------------------------------------------ 0

NC_045512.2 ACTGGGCATTGATTTAGATGAGTGGAGTATGGCTACATACTACTTATTTGATGAGTCTGG 3000

MT186676.1 ------------------------------------------------------------ 0

NC_045512.2 TGAGTTTAAATTGGCTTCACATATGTATTGTTCTTTCTACCCTCCAGATGAGGATGAAGA 3060

MT186676.1 ------------------------------------------------------------ 0

NC_045512.2 AGAAGGTGATTGTGAAGAAGAAGAGTTTGAGCCATCAACTCAATATGAGTATGGTACTGA 3120

MT186676.1 ------------------------------------------------------------ 0

NC_045512.2 AGATGATTACCAAGGTAAACCTTTGGAATTTGGTGCCACTTCTGCTGCTCTTCAACCTGA 3180

MT186676.1 ------------------------------------------------------------ 0

NC_045512.2 AGAAGAGCAAGAAGAAGATTGGTTAGATGATGATAGTCAACAAACTGTTGGTCAACAAGA 3240

MT186676.1 ------------------------------------------------------------ 0

NC_045512.2 CGGCAGTGAGGACAATCAGACAACTACTATTCAAACAATTGTTGAGGTTCAACCTCAATT 3300

MT186676.1 ------------------------------------------------------------ 0

NC_045512.2 AGAGATGGAACTTACACCAGTTGTTCAGACTATTGAAGTGAATAGTTTTAGTGGTTATTT 3360

MT186676.1 ------------------------------------------------------------ 0

NC_045512.2 AAAACTTACTGACAATGTATACATTAAAAATGCAGACATTGTGGAAGAAGCTAAAAAGGT 3420

MT186676.1 ------------------------------------------------------------ 0

NC_045512.2 AAAACCAACAGTGGTTGTTAATGCAGCCAATGTTTACCTTAAACATGGAGGAGGTGTTGC 3480

MT186676.1 ------------------------------------------------------------ 0

NC_045512.2 AGGAGCCTTAAATAAGGCTACTAACAATGCCATGCAAGTTGAATCTGATGATTACATAGC 3540

MT186676.1 ------------------------------------------------------------ 0

NC_045512.2 TACTAATGGACCACTTAAAGTGGGTGGTAGTTGTGTTTTAAGCGGACACAATCTTGCTAA 3600

MT186676.1 ------------------------------------------------------------ 0

NC_045512.2 ACACTGTCTTCATGTTGTCGGCCCAAATGTTAACAAAGGTGAAGACATTCAACTTCTTAA 3660

MT186676.1 ------------------------------------------------------------ 0

NC_045512.2 GAGTGCTTATGAAAATTTTAATCAGCACGAAGTTCTACTTGCACCATTATTATCAGCTGG 3720

MT186676.1 ------------------------------------------------------------ 0

NC_045512.2 TATTTTTGGTGCTGACCCTATACATTCTTTAAGAGTTTGTGTAGATACTGTTCGCACAAA 3780

MT186676.1 ------------------------------------------------------------ 0

NC_045512.2 TGTCTACTTAGCTGTCTTTGATAAAAATCTCTATGACAAACTTGTTTCAAGCTTTTTGGA 3840

MT186676.1 ------------------------------------------------------------ 0

NC_045512.2 AATGAAGAGTGAAAAGCAAGTTGAACAAAAGATCGCTGAGATTCCTAAAGAGGAAGTTAA 3900

MT186676.1 ------------------------------------------------------------ 0

NC_045512.2 GCCATTTATAACTGAAAGTAAACCTTCAGTTGAACAGAGAAAACAAGATGATAAGAAAAT 3960

MT186676.1 ------------------------------------------------------------ 0

NC_045512.2 CAAAGCTTGTGTTGAAGAAGTTACAACAACTCTGGAAGAAACTAAGTTCCTCACAGAAAA 4020

MT186676.1 ------------------------------------------------------------ 0

NC_045512.2 CTTGTTACTTTATATTGACATTAATGGCAATCTTCATCCAGATTCTGCCACTCTTGTTAG 4080

MT186676.1 ------------------------------------------------------------ 0

NC_045512.2 TGACATTGACATCACTTTCTTAAAGAAAGATGCTCCATATATAGTGGGTGATGTTGTTCA 4140

MT186676.1 ------------------------------------------------------------ 0

NC_045512.2 AGAGGGTGTTTTAACTGCTGTGGTTATACCTACTAAAAAGGCTGGTGGCACTACTGAAAT 4200

MT186676.1 ------------------------------------------------------------ 0

NC_045512.2 GCTAGCGAAAGCTTTGAGAAAAGTGCCAACAGACAATTATATAACCACTTACCCGGGTCA 4260

MT186676.1 ------------------------------------------------------------ 0

NC_045512.2 GGGTTTAAATGGTTACACTGTAGAGGAGGCAAAGACAGTGCTTAAAAAGTGTAAAAGTGC 4320

MT186676.1 ------------------------------------------------------------ 0

NC_045512.2 CTTTTACATTCTACCATCTATTATCTCTAATGAGAAGCAAGAAATTCTTGGAACTGTTTC 4380

MT186676.1 ------------------------------------------------------------ 0

NC_045512.2 TTGGAATTTGCGAGAAATGCTTGCACATGCAGAAGAAACACGCAAATTAATGCCTGTCTG 4440

MT186676.1 ------------------------------------------------------------ 0

NC_045512.2 TGTGGAAACTAAAGCCATAGTTTCAACTATACAGCGTAAATATAAGGGTATTAAAATACA 4500

MT186676.1 ------------------------------------------------------------ 0

NC_045512.2 AGAGGGTGTGGTTGATTATGGTGCTAGATTTTACTTTTACACCAGTAAAACAACTGTAGC 4560

MT186676.1 ------------------------------------------------------------ 0

NC_045512.2 GTCACTTATCAACACACTTAACGATCTAAATGAAACTCTTGTTACAATGCCACTTGGCTA 4620

MT186676.1 ------------------------------------------------------------ 0

NC_045512.2 TGTAACACATGGCTTAAATTTGGAAGAAGCTGCTCGGTATATGAGATCTCTCAAAGTGCC 4680

MT186676.1 ------------------------------------------------------------ 0

NC_045512.2 AGCTACAGTTTCTGTTTCTTCACCTGATGCTGTTACAGCGTATAATGGTTATCTTACTTC 4740

MT186676.1 ------------------------------------------------------------ 0

NC_045512.2 TTCTTCTAAAACACCTGAAGAACATTTTATTGAAACCATCTCACTTGCTGGTTCCTATAA 4800

MT186676.1 ------------------------------------------------------------ 0

NC_045512.2 AGATTGGTCCTATTCTGGACAATCTACACAACTAGGTATAGAATTTCTTAAGAGAGGTGA 4860

MT186676.1 ------------------------------------------------------------ 0

NC_045512.2 TAAAAGTGTATATTACACTAGTAATCCTACCACATTCCACCTAGATGGTGAAGTTATCAC 4920

MT186676.1 ------------------------------------------------------------ 0

NC_045512.2 CTTTGACAATCTTAAGACACTTCTTTCTTTGAGAGAAGTGAGGACTATTAAGGTGTTTAC 4980

MT186676.1 ------------------------------------------------------------ 0

NC_045512.2 AACAGTAGACAACATTAACCTCCACACGCAAGTTGTGGACATGTCAATGACATATGGACA 5040

MT186676.1 ------------------------------------------------------------ 0

NC_045512.2 ACAGTTTGGTCCAACTTATTTGGATGGAGCTGATGTTACTAAAATAAAACCTCATAATTC 5100

MT186676.1 ------------------------------------------------------------ 0

NC_045512.2 ACATGAAGGTAAAACATTTTATGTTTTACCTAATGATGACACTCTACGTGTTGAGGCTTT 5160

MT186676.1 ------------------------------------------------------------ 0

NC_045512.2 TGAGTACTACCACACAACTGATCCTAGTTTTCTGGGTAGGTACATGTCAGCATTAAATCA 5220

MT186676.1 ------------------------------------------------------------ 0

NC_045512.2 CACTAAAAAGTGGAAATACCCACAAGTTAATGGTTTAACTTCTATTAAATGGGCAGATAA 5280

MT186676.1 ------------------------------------------------------------ 0

NC_045512.2 CAACTGTTATCTTGCCACTGCATTGTTAACACTCCAACAAATAGAGTTGAAGTTTAATCC 5340

MT186676.1 ------------------------------------------------------------ 0

NC_045512.2 ACCTGCTCTACAAGATGCTTATTACAGAGCAAGGGCTGGTGAAGCTGCTAACTTTTGTGC 5400

MT186676.1 ------------------------------------------------------------ 0

NC_045512.2 ACTTATCTTAGCCTACTGTAATAAGACAGTAGGTGAGTTAGGTGATGTTAGAGAAACAAT 5460

MT186676.1 ------------------------------------------------------------ 0

NC_045512.2 GAGTTACTTGTTTCAACATGCCAATTTAGATTCTTGCAAAAGAGTCTTGAACGTGGTGTG 5520

MT186676.1 ------------------------------------------------------------ 0

NC_045512.2 TAAAACTTGTGGACAACAGCAGACAACCCTTAAGGGTGTAGAAGCTGTTATGTACATGGG 5580

MT186676.1 ------------------------------------------------------------ 0

NC_045512.2 CACACTTTCTTATGAACAATTTAAGAAAGGTGTTCAGATACCTTGTACGTGTGGTAAACA 5640

MT186676.1 ------------------------------------------------------------ 0

NC_045512.2 AGCTACAAAATATCTAGTACAACAGGAGTCACCTTTTGTTATGATGTCAGCACCACCTGC 5700

MT186676.1 ------------------------------------------------------------ 0

NC_045512.2 TCAGTATGAACTTAAGCATGGTACATTTACTTGTGCTAGTGAGTACACTGGTAATTACCA 5760

MT186676.1 ------------------------------------------------------------ 0

NC_045512.2 GTGTGGTCACTATAAACATATAACTTCTAAAGAAACTTTGTATTGCATAGACGGTGCTTT 5820

MT186676.1 ------------------------------------------------------------ 0

NC_045512.2 ACTTACAAAGTCCTCAGAATACAAAGGTCCTATTACGGATGTTTTCTACAAAGAAAACAG 5880

MT186676.1 ------------------------------------------------------------ 0

NC_045512.2 TTACACAACAACCATAAAACCAGTTACTTATAAATTGGATGGTGTTGTTTGTACAGAAAT 5940

MT186676.1 ------------------------------------------------------------ 0

NC_045512.2 TGACCCTAAGTTGGACAATTATTATAAGAAAGACAATTCTTATTTCACAGAGCAACCAAT 6000

MT186676.1 ------------------------------------------------------------ 0

NC_045512.2 TGATCTTGTACCAAACCAACCATATCCAAACGCAAGCTTCGATAATTTTAAGTTTGTATG 6060

MT186676.1 ------------------------------------------------------------ 0

NC_045512.2 TGATAATATCAAATTTGCTGATGATTTAAACCAGTTAACTGGTTATAAGAAACCTGCTTC 6120

MT186676.1 ------------------------------------------------------------ 0

NC_045512.2 AAGAGAGCTTAAAGTTACATTTTTCCCTGACTTAAATGGTGATGTGGTGGCTATTGATTA 6180

MT186676.1 ------------------------------------------------------------ 0

NC_045512.2 TAAACACTACACACCCTCTTTTAAGAAAGGAGCTAAATTGTTACATAAACCTATTGTTTG 6240

MT186676.1 ------------------------------------------------------------ 0

NC_045512.2 GCATGTTAACAATGCAACTAATAAAGCCACGTATAAACCAAATACCTGGTGTATACGTTG 6300

MT186676.1 ------------------------------------------------------------ 0

NC_045512.2 TCTTTGGAGCACAAAACCAGTTGAAACATCAAATTCGTTTGATGTACTGAAGTCAGAGGA 6360

MT186676.1 ------------------------------------------------------------ 0

NC_045512.2 CGCGCAGGGAATGGATAATCTTGCCTGCGAAGATCTAAAACCAGTCTCTGAAGAAGTAGT 6420

MT186676.1 ------------------------------------------------------------ 0

NC_045512.2 GGAAAATCCTACCATACAGAAAGACGTTCTTGAGTGTAATGTGAAAACTACCGAAGTTGT 6480

MT186676.1 ------------------------------------------------------------ 0

NC_045512.2 AGGAGACATTATACTTAAACCAGCAAATAATAGTTTAAAAATTACAGAAGAGGTTGGCCA 6540

MT186676.1 ------------------------------------------------------------ 0

NC_045512.2 CACAGATCTAATGGCTGCTTATGTAGACAATTCTAGTCTTACTATTAAGAAACCTAATGA 6600

MT186676.1 ------------------------------------------------------------ 0

NC_045512.2 ATTATCTAGAGTATTAGGTTTGAAAACCCTTGCTACTCATGGTTTAGCTGCTGTTAATAG 6660

MT186676.1 ------------------------------------------------------------ 0

NC_045512.2 TGTCCCTTGGGATACTATAGCTAATTATGCTAAGCCTTTTCTTAACAAAGTTGTTAGTAC 6720

MT186676.1 ------------------------------------------------------------ 0

NC_045512.2 AACTACTAACATAGTTACACGGTGTTTAAACCGTGTTTGTACTAATTATATGCCTTATTT 6780

MT186676.1 ------------------------------------------------------------ 0

NC_045512.2 CTTTACTTTATTGCTACAATTGTGTACTTTTACTAGAAGTACAAATTCTAGAATTAAAGC 6840

MT186676.1 ------------------------------------------------------------ 0

NC_045512.2 ATCTATGCCGACTACTATAGCAAAGAATACTGTTAAGAGTGTCGGTAAATTTTGTCTAGA 6900

MT186676.1 ------------------------------------------------------------ 0

NC_045512.2 GGCTTCATTTAATTATTTGAAGTCACCTAATTTTTCTAAACTGATAAATATTATAATTTG 6960

MT186676.1 ------------------------------------------------------------ 0

NC_045512.2 GTTTTTACTATTAAGTGTTTGCCTAGGTTCTTTAATCTACTCAACCGCTGCTTTAGGTGT 7020

MT186676.1 ------------------------------------------------------------ 0

NC_045512.2 TTTAATGTCTAATTTAGGCATGCCTTCTTACTGTACTGGTTACAGAGAAGGCTATTTGAA 7080

MT186676.1 ------------------------------------------------------------ 0

NC_045512.2 CTCTACTAATGTCACTATTGCAACCTACTGTACTGGTTCTATACCTTGTAGTGTTTGTCT 7140

MT186676.1 ------------------------------------------------------------ 0

NC_045512.2 TAGTGGTTTAGATTCTTTAGACACCTATCCTTCTTTAGAAACTATACAAATTACCATTTC 7200

MT186676.1 ------------------------------------------------------------ 0

NC_045512.2 ATCTTTTAAATGGGATTTAACTGCTTTTGGCTTAGTTGCAGAGTGGTTTTTGGCATATAT 7260

MT186676.1 ------------------------------------------------------------ 0

NC_045512.2 TCTTTTCACTAGGTTTTTCTATGTACTTGGATTGGCTGCAATCATGCAATTGTTTTTCAG 7320

MT186676.1 ------------------------------------------------------------ 0

NC_045512.2 CTATTTTGCAGTACATTTTATTAGTAATTCTTGGCTTATGTGGTTAATAATTAATCTTGT 7380

MT186676.1 ------------------------------------------------------------ 0

NC_045512.2 ACAAATGGCCCCGATTTCAGCTATGGTTAGAATGTACATCTTCTTTGCATCATTTTATTA 7440

MT186676.1 ------------------------------------------------------------ 0

NC_045512.2 TGTATGGAAAAGTTATGTGCATGTTGTAGACGGTTGTAATTCATCAACTTGTATGATGTG 7500

MT186676.1 ------------------------------------------------------------ 0

NC_045512.2 TTACAAACGTAATAGAGCAACAAGAGTCGAATGTACAACTATTGTTAATGGTGTTAGAAG 7560

MT186676.1 ------------------------------------------------------------ 0

NC_045512.2 GTCCTTTTATGTCTATGCTAATGGAGGTAAAGGCTTTTGCAAACTACACAATTGGAATTG 7620

MT186676.1 ------------------------------------------------------------ 0

NC_045512.2 TGTTAATTGTGATACATTCTGTGCTGGTAGTACATTTATTAGTGATGAAGTTGCGAGAGA 7680

MT186676.1 ------------------------------------------------------------ 0

NC_045512.2 CTTGTCACTACAGTTTAAAAGACCAATAAATCCTACTGACCAGTCTTCTTACATCGTTGA 7740

MT186676.1 ------------------------------------------------------------ 0

NC_045512.2 TAGTGTTACAGTGAAGAATGGTTCCATCCATCTTTACTTTGATAAAGCTGGTCAAAAGAC 7800

MT186676.1 ------------------------------------------------------------ 0

NC_045512.2 TTATGAAAGACATTCTCTCTCTCATTTTGTTAACTTAGACAACCTGAGAGCTAATAACAC 7860

MT186676.1 ------------------------------------------------------------ 0

NC_045512.2 TAAAGGTTCATTGCCTATTAATGTTATAGTTTTTGATGGTAAATCAAAATGTGAAGAATC 7920

MT186676.1 ------------------------------------------------------------ 0

NC_045512.2 ATCTGCAAAATCAGCGTCTGTTTACTACAGTCAGCTTATGTGTCAACCTATACTGTTACT 7980

MT186676.1 ------------------------------------------------------------ 0

NC_045512.2 AGATCAGGCATTAGTGTCTGATGTTGGTGATAGTGCGGAAGTTGCAGTTAAAATGTTTGA 8040

MT186676.1 ------------------------------------------------------------ 0

NC_045512.2 TGCTTACGTTAATACGTTTTCATCAACTTTTAACGTACCAATGGAAAAACTCAAAACACT 8100

MT186676.1 ------------------------------------------------------------ 0

NC_045512.2 AGTTGCAACTGCAGAAGCTGAACTTGCAAAGAATGTGTCCTTAGACAATGTCTTATCTAC 8160

MT186676.1 ------------------------------------------------------------ 0

NC_045512.2 TTTTATTTCAGCAGCTCGGCAAGGGTTTGTTGATTCAGATGTAGAAACTAAAGATGTTGT 8220

MT186676.1 ------------------------------------------------------------ 0

NC_045512.2 TGAATGTCTTAAATTGTCACATCAATCTGACATAGAAGTTACTGGCGATAGTTGTAATAA 8280

MT186676.1 ------------------------------------------------------------ 0

NC_045512.2 CTATATGCTCACCTATAACAAAGTTGAAAACATGACACCCCGTGACCTTGGTGCTTGTAT 8340

MT186676.1 ------------------------------------------------------------ 0

NC_045512.2 TGACTGTAGTGCGCGTCATATTAATGCGCAGGTAGCAAAAAGTCACAACATTGCTTTGAT 8400

MT186676.1 ------------------------------------------------------------ 0

NC_045512.2 ATGGAACGTTAAAGATTTCATGTCATTGTCTGAACAACTACGAAAACAAATACGTAGTGC 8460

MT186676.1 ------------------------------------------------------------ 0

NC_045512.2 TGCTAAAAAGAATAACTTACCTTTTAAGTTGACATGTGCAACTACTAGACAAGTTGTTAA 8520

MT186676.1 ------------------------------------------------------------ 0

NC_045512.2 TGTTGTAACAACAAAGATAGCACTTAAGGGTGGTAAAATTGTTAATAATTGGTTGAAGCA 8580

MT186676.1 ------------------------------------------------------------ 0

NC_045512.2 GTTAATTAAAGTTACACTTGTGTTCCTTTTTGTTGCTGCTATTTTCTATTTAATAACACC 8640

MT186676.1 ------------------------------------------------------------ 0

NC_045512.2 TGTTCATGTCATGTCTAAACATACTGACTTTTCAAGTGAAATCATAGGATACAAGGCTAT 8700

MT186676.1 ------------------------------------------------------------ 0

NC_045512.2 TGATGGTGGTGTCACTCGTGACATAGCATCTACAGATACTTGTTTTGCTAACAAACATGC 8760

MT186676.1 ------------------------------------------------------------ 0

NC_045512.2 TGATTTTGACACATGGTTTAGCCAGCGTGGTGGTAGTTATACTAATGACAAAGCTTGCCC 8820

MT186676.1 ------------------------------------------------------------ 0

NC_045512.2 ATTGATTGCTGCAGTCATAACAAGAGAAGTGGGTTTTGTCGTGCCTGGTTTGCCTGGCAC 8880

MT186676.1 ------------------------------------------------------------ 0

NC_045512.2 GATATTACGCACAACTAATGGTGACTTTTTGCATTTCTTACCTAGAGTTTTTAGTGCAGT 8940

MT186676.1 ------------------------------------------------------------ 0

NC_045512.2 TGGTAACATCTGTTACACACCATCAAAACTTATAGAGTACACTGACTTTGCAACATCAGC 9000

MT186676.1 ------------------------------------------------------------ 0

NC_045512.2 TTGTGTTTTGGCTGCTGAATGTACAATTTTTAAAGATGCTTCTGGTAAGCCAGTACCATA 9060

MT186676.1 ------------------------------------------------------------ 0

NC_045512.2 TTGTTATGATACCAATGTACTAGAAGGTTCTGTTGCTTATGAAAGTTTACGCCCTGACAC 9120

MT186676.1 ------------------------------------------------------------ 0

NC_045512.2 ACGTTATGTGCTCATGGATGGCTCTATTATTCAATTTCCTAACACCTACCTTGAAGGTTC 9180

MT186676.1 ------------------------------------------------------------ 0

NC_045512.2 TGTTAGAGTGGTAACAACTTTTGATTCTGAGTACTGTAGGCACGGCACTTGTGAAAGATC 9240

MT186676.1 ------------------------------------------------------------ 0

NC_045512.2 AGAAGCTGGTGTTTGTGTATCTACTAGTGGTAGATGGGTACTTAACAATGATTATTACAG 9300

MT186676.1 ------------------------------------------------------------ 0

NC_045512.2 ATCTTTACCAGGAGTTTTCTGTGGTGTAGATGCTGTAAATTTACTTACTAATATGTTTAC 9360

MT186676.1 ------------------------------------------------------------ 0

NC_045512.2 ACCACTAATTCAACCTATTGGTGCTTTGGACATATCAGCATCTATAGTAGCTGGTGGTAT 9420

MT186676.1 ------------------------------------------------------------ 0

NC_045512.2 TGTAGCTATCGTAGTAACATGCCTTGCCTACTATTTTATGAGGTTTAGAAGAGCTTTTGG 9480

MT186676.1 ------------------------------------------------------------ 0

NC_045512.2 TGAATACAGTCATGTAGTTGCCTTTAATACTTTACTATTCCTTATGTCATTCACTGTACT 9540

MT186676.1 ------------------------------------------------------------ 0

NC_045512.2 CTGTTTAACACCAGTTTACTCATTCTTACCTGGTGTTTATTCTGTTATTTACTTGTACTT 9600

MT186676.1 ------------------------------------------------------------ 0

NC_045512.2 GACATTTTATCTTACTAATGATGTTTCTTTTTTAGCACATATTCAGTGGATGGTTATGTT 9660

MT186676.1 ------------------------------------------------------------ 0

NC_045512.2 CACACCTTTAGTACCTTTCTGGATAACAATTGCTTATATCATTTGTATTTCCACAAAGCA 9720

MT186676.1 ------------------------------------------------------------ 0

NC_045512.2 TTTCTATTGGTTCTTTAGTAATTACCTAAAGAGACGTGTAGTCTTTAATGGTGTTTCCTT 9780

MT186676.1 ------------------------------------------------------------ 0

NC_045512.2 TAGTACTTTTGAAGAAGCTGCGCTGTGCACCTTTTTGTTAAATAAAGAAATGTATCTAAA 9840

MT186676.1 ------------------------------------------------------------ 0

NC_045512.2 GTTGCGTAGTGATGTGCTATTACCTCTTACGCAATATAATAGATACTTAGCTCTTTATAA 9900

MT186676.1 ------------------------------------------------------------ 0

NC_045512.2 TAAGTACAAGTATTTTAGTGGAGCAATGGATACAACTAGCTACAGAGAAGCTGCTTGTTG 9960

MT186676.1 ------------------------------------------------------------ 0

NC_045512.2 TCATCTCGCAAAGGCTCTCAATGACTTCAGTAACTCAGGTTCTGATGTTCTTTACCAACC 10020

MT186676.1 ------------------------------------------------------------ 0

NC_045512.2 ACCACAAACCTCTATCACCTCAGCTGTTTTGCAGAGTGGTTTTAGAAAAATGGCATTCCC 10080

MT186676.1 ------------------------------------------------------------ 0

NC_045512.2 ATCTGGTAAAGTTGAGGGTTGTATGGTACAAGTAACTTGTGGTACAACTACACTTAACGG 10140

MT186676.1 ------------------------------------------------------------ 0

NC_045512.2 TCTTTGGCTTGATGACGTAGTTTACTGTCCAAGACATGTGATCTGCACCTCTGAAGACAT 10200

MT186676.1 ------------------------------------------------------------ 0

NC_045512.2 GCTTAACCCTAATTATGAAGATTTACTCATTCGTAAGTCTAATCATAATTTCTTGGTACA 10260

MT186676.1 ------------------------------------------------------------ 0

NC_045512.2 GGCTGGTAATGTTCAACTCAGGGTTATTGGACATTCTATGCAAAATTGTGTACTTAAGCT 10320

MT186676.1 ------------------------------------------------------------ 0

NC_045512.2 TAAGGTTGATACAGCCAATCCTAAGACACCTAAGTATAAGTTTGTTCGCATTCAACCAGG 10380

MT186676.1 ------------------------------------------------------------ 0

NC_045512.2 ACAGACTTTTTCAGTGTTAGCTTGTTACAATGGTTCACCATCTGGTGTTTACCAATGTGC 10440

MT186676.1 ------------------------------------------------------------ 0

NC_045512.2 TATGAGGCCCAATTTCACTATTAAGGGTTCATTCCTTAATGGTTCATGTGGTAGTGTTGG 10500

MT186676.1 ------------------------------------------------------------ 0

NC_045512.2 TTTTAACATAGATTATGACTGTGTCTCTTTTTGTTACATGCACCATATGGAATTACCAAC 10560

MT186676.1 ------------------------------------------------------------ 0

NC_045512.2 TGGAGTTCATGCTGGCACAGACTTAGAAGGTAACTTTTATGGACCTTTTGTTGACAGGCA 10620

MT186676.1 ------------------------------------------------------------ 0

NC_045512.2 AACAGCACAAGCAGCTGGTACGGACACAACTATTACAGTTAATGTTTTAGCTTGGTTGTA 10680

MT186676.1 ------------------------------------------------------------ 0

NC_045512.2 CGCTGCTGTTATAAATGGAGACAGGTGGTTTCTCAATCGATTTACCACAACTCTTAATGA 10740

MT186676.1 ------------------------------------------------------------ 0

NC_045512.2 CTTTAACCTTGTGGCTATGAAGTACAATTATGAACCTCTAACACAAGACCATGTTGACAT 10800

MT186676.1 ------------------------------------------------------------ 0

NC_045512.2 ACTAGGACCTCTTTCTGCTCAAACTGGAATTGCCGTTTTAGATATGTGTGCTTCATTAAA 10860

MT186676.1 ------------------------------------------------------------ 0

NC_045512.2 AGAATTACTGCAAAATGGTATGAATGGACGTACCATATTGGGTAGTGCTTTATTAGAAGA 10920

MT186676.1 ------------------------------------------------------------ 0

NC_045512.2 TGAATTTACACCTTTTGATGTTGTTAGACAATGCTCAGGTGTTACTTTCCAAAGTGCAGT 10980

MT186676.1 ------------------------------------------------------------ 0

NC_045512.2 GAAAAGAACAATCAAGGGTACACACCACTGGTTGTTACTCACAATTTTGACTTCACTTTT 11040

MT186676.1 ------------------------------------------------------------ 0

NC_045512.2 AGTTTTAGTCCAGAGTACTCAATGGTCTTTGTTCTTTTTTTTGTATGAAAATGCCTTTTT 11100

MT186676.1 ------------------------------------------------------------ 0

NC_045512.2 ACCTTTTGCTATGGGTATTATTGCTATGTCTGCTTTTGCAATGATGTTTGTCAAACATAA 11160

MT186676.1 ------------------------------------------------------------ 0

NC_045512.2 GCATGCATTTCTCTGTTTGTTTTTGTTACCTTCTCTTGCCACTGTAGCTTATTTTAATAT 11220

MT186676.1 ------------------------------------------------------------ 0

NC_045512.2 GGTCTATATGCCTGCTAGTTGGGTGATGCGTATTATGACATGGTTGGATATGGTTGATAC 11280

MT186676.1 ------------------------------------------------------------ 0

NC_045512.2 TAGTTTGTCTGGTTTTAAGCTAAAAGACTGTGTTATGTATGCATCAGCTGTAGTGTTACT 11340

MT186676.1 ------------------------------------------------------------ 0

NC_045512.2 AATCCTTATGACAGCAAGAACTGTGTATGATGATGGTGCTAGGAGAGTGTGGACACTTAT 11400

MT186676.1 ------------------------------------------------------------ 0

NC_045512.2 GAATGTCTTGACACTCGTTTATAAAGTTTATTATGGTAATGCTTTAGATCAAGCCATTTC 11460

MT186676.1 ------------------------------------------------------------ 0

NC_045512.2 CATGTGGGCTCTTATAATCTCTGTTACTTCTAACTACTCAGGTGTAGTTACAACTGTCAT 11520

MT186676.1 ------------------------------------------------------------ 0

NC_045512.2 GTTTTTGGCCAGAGGTATTGTTTTTATGTGTGTTGAGTATTGCCCTATTTTCTTCATAAC 11580

MT186676.1 ------------------------------------------------------------ 0

NC_045512.2 TGGTAATACACTTCAGTGTATAATGCTAGTTTATTGTTTCTTAGGCTATTTTTGTACTTG 11640

MT186676.1 ------------------------------------------------------------ 0

NC_045512.2 TTACTTTGGCCTCTTTTGTTTACTCAACCGCTACTTTAGACTGACTCTTGGTGTTTATGA 11700

MT186676.1 ------------------------------------------------------------ 0

NC_045512.2 TTACTTAGTTTCTACACAGGAGTTTAGATATATGAATTCACAGGGACTACTCCCACCCAA 11760

MT186676.1 ------------------------------------------------------------ 0

NC_045512.2 GAATAGCATAGATGCCTTCAAACTCAACATTAAATTGTTGGGTGTTGGTGGCAAACCTTG 11820

MT186676.1 ------------------------------------------------------------ 0

NC_045512.2 TATCAAAGTAGCCACTGTACAGTCTAAAATGTCAGATGTAAAGTGCACATCAGTAGTCTT 11880

MT186676.1 ------------------------------------------------------------ 0

NC_045512.2 ACTCTCAGTTTTGCAACAACTCAGAGTAGAATCATCATCTAAATTGTGGGCTCAATGTGT 11940

MT186676.1 ------------------------------------------------------------ 0

NC_045512.2 CCAGTTACACAATGACATTCTCTTAGCTAAAGATACTACTGAAGCCTTTGAAAAAATGGT 12000

MT186676.1 ------------------------------------------------------------ 0

NC_045512.2 TTCACTACTTTCTGTTTTGCTTTCCATGCAGGGTGCTGTAGACATAAACAAGCTTTGTGA 12060

MT186676.1 ------------------------------------------------------------ 0

NC_045512.2 AGAAATGCTGGACAACAGGGCAACCTTACAAGCTATAGCCTCAGAGTTTAGTTCCCTTCC 12120

MT186676.1 ------------------------------------------------------------ 0

NC_045512.2 ATCATATGCAGCTTTTGCTACTGCTCAAGAAGCTTATGAGCAGGCTGTTGCTAATGGTGA 12180

MT186676.1 ------------------------------------------------------------ 0

NC_045512.2 TTCTGAAGTTGTTCTTAAAAAGTTGAAGAAGTCTTTGAATGTGGCTAAATCTGAATTTGA 12240

MT186676.1 ------------------------------------------------------------ 0

NC_045512.2 CCGTGATGCAGCCATGCAACGTAAGTTGGAAAAGATGGCTGATCAAGCTATGACCCAAAT 12300

MT186676.1 ------------------------------------------------------------ 0

NC_045512.2 GTATAAACAGGCTAGATCTGAGGACAAGAGGGCAAAAGTTACTAGTGCTATGCAGACAAT 12360

MT186676.1 ------------------------------------------------------------ 0

NC_045512.2 GCTTTTCACTATGCTTAGAAAGTTGGATAATGATGCACTCAACAACATTATCAACAATGC 12420

MT186676.1 ------------------------------------------------------------ 0

NC_045512.2 AAGAGATGGTTGTGTTCCCTTGAACATAATACCTCTTACAACAGCAGCCAAACTAATGGT 12480

MT186676.1 ------------------------------------------------------------ 0

NC_045512.2 TGTCATACCAGACTATAACACATATAAAAATACGTGTGATGGTACAACATTTACTTATGC 12540

MT186676.1 ------------------------------------------------------------ 0

NC_045512.2 ATCAGCATTGTGGGAAATCCAACAGGTTGTAGATGCAGATAGTAAAATTGTTCAACTTAG 12600

MT186676.1 ------------------------------------------------------------ 0

NC_045512.2 TGAAATTAGTATGGACAATTCACCTAATTTAGCATGGCCTCTTATTGTAACAGCTTTAAG 12660

MT186676.1 ------------------------------------------------------------ 0

NC_045512.2 GGCCAATTCTGCTGTCAAATTACAGAATAATGAGCTTAGTCCTGTTGCACTACGACAGAT 12720

MT186676.1 ------------------------------------------------------------ 0

NC_045512.2 GTCTTGTGCTGCCGGTACTACACAAACTGCTTGCACTGATGACAATGCGTTAGCTTACTA 12780

MT186676.1 ------------------------------------------------------------ 0

NC_045512.2 CAACACAACAAAGGGAGGTAGGTTTGTACTTGCACTGTTATCCGATTTACAGGATTTGAA 12840

MT186676.1 ------------------------------------------------------------ 0

NC_045512.2 ATGGGCTAGATTCCCTAAGAGTGATGGAACTGGTACTATCTATACAGAACTGGAACCACC 12900

MT186676.1 ------------------------------------------------------------ 0

NC_045512.2 TTGTAGGTTTGTTACAGACACACCTAAAGGTCCTAAAGTGAAGTATTTATACTTTATTAA 12960

MT186676.1 ------------------------------------------------------------ 0

NC_045512.2 AGGATTAAACAACCTAAATAGAGGTATGGTACTTGGTAGTTTAGCTGCCACAGTACGTCT 13020

MT186676.1 ------------------------------------------------------------ 0

NC_045512.2 ACAAGCTGGTAATGCAACAGAAGTGCCTGCCAATTCAACTGTATTATCTTTCTGTGCTTT 13080

MT186676.1 ------------------------------------------------------------ 0

NC_045512.2 TGCTGTAGATGCTGCTAAAGCTTACAAAGATTATCTAGCTAGTGGGGGACAACCAATCAC 13140

MT186676.1 ------------------------------------------------------------ 0

NC_045512.2 TAATTGTGTTAAGATGTTGTGTACACACACTGGTACTGGTCAGGCAATAACAGTTACACC 13200

MT186676.1 ------------------------------------------------------------ 0

NC_045512.2 GGAAGCCAATATGGATCAAGAATCCTTTGGTGGTGCATCGTGTTGTCTGTACTGCCGTTG 13260

MT186676.1 ------------------------------------------------------------ 0

NC_045512.2 CCACATAGATCATCCAAATCCTAAAGGATTTTGTGACTTAAAAGGTAAGTATGTACAAAT 13320

MT186676.1 ------------------------------------------------------------ 0

NC_045512.2 ACCTACAACTTGTGCTAATGACCCTGTGGGTTTTACACTTAAAAACACAGTCTGTACCGT 13380

MT186676.1 ------------------------------------------------------------ 0

NC_045512.2 CTGCGGTATGTGGAAAGGTTATGGCTGTAGTTGTGATCAACTCCGCGAACCCATGCTTCA 13440

MT186676.1 ------------------------------------------------------------ 0

NC_045512.2 GTCAGCTGATGCACAATCGTTTTTAAACGGGTTTGCGGTGTAAGTGCAGCCCGTCTTACA 13500

MT186676.1 ------------------------------------------------------------ 0

NC_045512.2 CCGTGCGGCACAGGCACTAGTACTGATGTCGTATACAGGGCTTTTGACATCTACAATGAT 13560

MT186676.1 ------------------------------------------------------------ 0

NC_045512.2 AAAGTAGCTGGTTTTGCTAAATTCCTAAAAACTAATTGTTGTCGCTTCCAAGAAAAGGAC 13620

MT186676.1 ------------------------------------------------------------ 0

NC_045512.2 GAAGATGACAATTTAATTGATTCTTACTTTGTAGTTAAGAGACACACTTTCTCTAACTAC 13680

MT186676.1 ------------------------------------------------------------ 0

NC_045512.2 CAACATGAAGAAACAATTTATAATTTACTTAAGGATTGTCCAGCTGTTGCTAAACATGAC 13740

MT186676.1 ------------------------------------------------------------ 0

NC_045512.2 TTCTTTAAGTTTAGAATAGACGGTGACATGGTACCACATATATCACGTCAACGTCTTACT 13800

MT186676.1 ------------------------------------------------------------ 0

NC_045512.2 AAATACACAATGGCAGACCTCGTCTATGCTTTAAGGCATTTTGATGAAGGTAATTGTGAC 13860

MT186676.1 ------------------------------------------------------------ 0

NC_045512.2 ACATTAAAAGAAATACTTGTCACATACAATTGTTGTGATGATGATTATTTCAATAAAAAG 13920

MT186676.1 ------------------------------------------------------------ 0

NC_045512.2 GACTGGTATGATTTTGTAGAAAACCCAGATATATTACGCGTATACGCCAACTTAGGTGAA 13980

MT186676.1 ------------------------------------------------------------ 0

NC_045512.2 CGTGTACGCCAAGCTTTGTTAAAAACAGTACAATTCTGTGATGCCATGCGAAATGCTGGT 14040

MT186676.1 ------------------------------------------------------------ 0

NC_045512.2 ATTGTTGGTGTACTGACATTAGATAATCAAGATCTCAATGGTAACTGGTATGATTTCGGT 14100

MT186676.1 ------------------------------------------------------------ 0

NC_045512.2 GATTTCATACAAACCACGCCAGGTAGTGGAGTTCCTGTTGTAGATTCTTATTATTCATTG 14160

MT186676.1 ------------------------------------------------------------ 0

NC_045512.2 TTAATGCCTATATTAACCTTGACCAGGGCTTTAACTGCAGAGTCACATGTTGACACTGAC 14220

MT186676.1 ------------------------------------------------------------ 0

NC_045512.2 TTAACAAAGCCTTACATTAAGTGGGATTTGTTAAAATATGACTTCACGGAAGAGAGGTTA 14280

MT186676.1 ------------------------------------------------------------ 0

NC_045512.2 AAACTCTTTGACCGTTATTTTAAATATTGGGATCAGACATACCACCCAAATTGTGTTAAC 14340

MT186676.1 ------------------------------------------------------------ 0

NC_045512.2 TGTTTGGATGACAGATGCATTCTGCATTGTGCAAACTTTAATGTTTTATTCTCTACAGTG 14400

MT186676.1 ------------------------------------------------------------ 0

NC_045512.2 TTCCCACCTACAAGTTTTGGACCACTAGTGAGAAAAATATTTGTTGATGGTGTTCCATTT 14460

MT186676.1 ------------------------------------------------------------ 0

NC_045512.2 GTAGTTTCAACTGGATACCACTTCAGAGAGCTAGGTGTTGTACATAATCAGGATGTAAAC 14520

MT186676.1 ------------------------------------------------------------ 0

NC_045512.2 TTACATAGCTCTAGACTTAGTTTTAAGGAATTACTTGTGTATGCTGCTGACCCTGCTATG 14580

MT186676.1 ------------------------------------------------------------ 0

NC_045512.2 CACGCTGCTTCTGGTAATCTATTACTAGATAAACGCACTACGTGCTTTTCAGTAGCTGCA 14640

MT186676.1 ------------------------------------------------------------ 0

NC_045512.2 CTTACTAACAATGTTGCTTTTCAAACTGTCAAACCCGGTAATTTTAACAAAGACTTCTAT 14700

MT186676.1 ------------------------------------------------------------ 0

NC_045512.2 GACTTTGCTGTGTCTAAGGGTTTCTTTAAGGAAGGAAGTTCTGTTGAATTAAAACACTTC 14760

MT186676.1 ------------------------------------------------------------ 0

NC_045512.2 TTCTTTGCTCAGGATGGTAATGCTGCTATCAGCGATTATGACTACTATCGTTATAATCTA 14820

MT186676.1 ------------------------------------------------------------ 0

NC_045512.2 CCAACAATGTGTGATATCAGACAACTACTATTTGTAGTTGAAGTTGTTGATAAGTACTTT 14880

MT186676.1 ------------------------------------------------------------ 0

NC_045512.2 GATTGTTACGATGGTGGCTGTATTAATGCTAACCAAGTCATCGTCAACAACCTAGACAAA 14940

MT186676.1 ------------------------------------------------------------ 0

NC_045512.2 TCAGCTGGTTTTCCATTTAATAAATGGGGTAAGGCTAGACTTTATTATGATTCAATGAGT 15000

MT186676.1 ------------------------------------------------------------ 0

NC_045512.2 TATGAGGATCAAGATGCACTTTTCGCATATACAAAACGTAATGTCATCCCTACTATAACT 15060

MT186676.1 ------------------------------------------------------------ 0

NC_045512.2 CAAATGAATCTTAAGTATGCCATTAGTGCAAAGAATAGAGCTCGCACCGTAGCTGGTGTC 15120

MT186676.1 ------------------------------------------------------------ 0

NC_045512.2 TCTATCTGTAGTACTATGACCAATAGACAGTTTCATCAAAAATTATTGAAATCAATAGCC 15180

MT186676.1 ------------------------------------------------------------ 0

NC_045512.2 GCCACTAGAGGAGCTACTGTAGTAATTGGAACAAGCAAATTCTATGGTGGTTGGCACAAC 15240

MT186676.1 ------------------------------------------------------------ 0

NC_045512.2 ATGTTAAAAACTGTTTATAGTGATGTAGAAAACCCTCACCTTATGGGTTGGGATTATCCT 15300

MT186676.1 ------------------------------------------------------------ 0

NC_045512.2 AAATGTGATAGAGCCATGCCTAACATGCTTAGAATTATGGCCTCACTTGTTCTTGCTCGC 15360

MT186676.1 ------------------------------------------------------------ 0

NC_045512.2 AAACATACAACGTGTTGTAGCTTGTCACACCGTTTCTATAGATTAGCTAATGAGTGTGCT 15420

MT186676.1 ------------------------------------------------------------ 0

NC_045512.2 CAAGTATTGAGTGAAATGGTCATGTGTGGCGGTTCACTATATGTTAAACCAGGTGGAACC 15480

MT186676.1 ------------------------------------------------------------ 0

NC_045512.2 TCATCAGGAGATGCCACAACTGCTTATGCTAATAGTGTTTTTAACATTTGTCAAGCTGTC 15540

MT186676.1 ------------------------------------------------------------ 0

NC_045512.2 ACGGCCAATGTTAATGCACTTTTATCTACTGATGGTAACAAAATTGCCGATAAGTATGTC 15600

MT186676.1 ------------------------------------------------------------ 0

NC_045512.2 CGCAATTTACAACACAGACTTTATGAGTGTCTCTATAGAAATAGAGATGTTGACACAGAC 15660

MT186676.1 ------------------------------------------------------------ 0

NC_045512.2 TTTGTGAATGAGTTTTACGCATATTTGCGTAAACATTTCTCAATGATGATACTCTCTGAC 15720

MT186676.1 ------------------------------------------------------------ 0

NC_045512.2 GATGCTGTTGTGTGTTTCAATAGCACTTATGCATCTCAAGGTCTAGTGGCTAGCATAAAG 15780

MT186676.1 ------------------------------------------------------------ 0

NC_045512.2 AACTTTAAGTCAGTTCTTTATTATCAAAACAATGTTTTTATGTCTGAAGCAAAATGTTGG 15840

MT186676.1 ------------------------------------------------------------ 0

NC_045512.2 ACTGAGACTGACCTTACTAAAGGACCTCATGAATTTTGCTCTCAACATACAATGCTAGTT 15900

MT186676.1 ------------------------------------------------------------ 0

NC_045512.2 AAACAGGGTGATGATTATGTGTACCTTCCTTACCCAGATCCATCAAGAATCCTAGGGGCC 15960

MT186676.1 ------------------------------------------------------------ 0

NC_045512.2 GGCTGTTTTGTAGATGATATCGTAAAAACAGATGGTACACTTATGATTGAACGGTTCGTG 16020

MT186676.1 ------------------------------------------------------------ 0

NC_045512.2 TCTTTAGCTATAGATGCTTACCCACTTACTAAACATCCTAATCAGGAGTATGCTGATGTC 16080

MT186676.1 ------------------------------------------------------------ 0

NC_045512.2 TTTCATTTGTACTTACAATACATAAGAAAGCTACATGATGAGTTAACAGGACACATGTTA 16140

MT186676.1 ------------------------------------------------------------ 0

NC_045512.2 GACATGTATTCTGTTATGCTTACTAATGATAACACTTCAAGGTATTGGGAACCTGAGTTT 16200

MT186676.1 ------------------------------------------------------------ 0

NC_045512.2 TATGAGGCTATGTACACACCGCATACAGTCTTACAGGCTGTTGGGGCTTGTGTTCTTTGC 16260

MT186676.1 ------------------------------------------------------------ 0

NC_045512.2 AATTCACAGACTTCATTAAGATGTGGTGCTTGCATACGTAGACCATTCTTATGTTGTAAA 16320

MT186676.1 ------------------------------------------------------------ 0

NC_045512.2 TGCTGTTACGACCATGTCATATCAACATCACATAAATTAGTCTTGTCTGTTAATCCGTAT 16380

MT186676.1 ------------------------------------------------------------ 0

NC_045512.2 GTTTGCAATGCTCCAGGTTGTGATGTCACAGATGTGACTCAACTTTACTTAGGAGGTATG 16440

MT186676.1 ------------------------------------------------------------ 0

NC_045512.2 AGCTATTATTGTAAATCACATAAACCACCCATTAGTTTTCCATTGTGTGCTAATGGACAA 16500

MT186676.1 ------------------------------------------------------------ 0

NC_045512.2 GTTTTTGGTTTATATAAAAATACATGTGTTGGTAGCGATAATGTTACTGACTTTAATGCA 16560

MT186676.1 ------------------------------------------------------------ 0

NC_045512.2 ATTGCAACATGTGACTGGACAAATGCTGGTGATTACATTTTAGCTAACACCTGTACTGAA 16620

MT186676.1 ------------------------------------------------------------ 0

NC_045512.2 AGACTCAAGCTTTTTGCAGCAGAAACGCTCAAAGCTACTGAGGAGACATTTAAACTGTCT 16680

MT186676.1 ------------------------------------------------------------ 0

NC_045512.2 TATGGTATTGCTACTGTACGTGAAGTGCTGTCTGACAGAGAATTACATCTTTCATGGGAA 16740

MT186676.1 ------------------------------------------------------------ 0

NC_045512.2 GTTGGTAAACCTAGACCACCACTTAACCGAAATTATGTCTTTACTGGTTATCGTGTAACT 16800

MT186676.1 ------------------------------------------------------------ 0

NC_045512.2 AAAAACAGTAAAGTACAAATAGGAGAGTACACCTTTGAAAAAGGTGACTATGGTGATGCT 16860

MT186676.1 ------------------------------------------------------------ 0

NC_045512.2 GTTGTTTACCGAGGTACAACAACTTACAAATTAAATGTTGGTGATTATTTTGTGCTGACA 16920

MT186676.1 ------------------------------------------------------------ 0

NC_045512.2 TCACATACAGTAATGCCATTAAGTGCACCTACACTAGTGCCACAAGAGCACTATGTTAGA 16980

MT186676.1 ------------------------------------------------------------ 0

NC_045512.2 ATTACTGGCTTATACCCAACACTCAATATCTCAGATGAGTTTTCTAGCAATGTTGCAAAT 17040

MT186676.1 ------------------------------------------------------------ 0

NC_045512.2 TATCAAAAGGTTGGTATGCAAAAGTATTCTACACTCCAGGGACCACCTGGTACTGGTAAG 17100

MT186676.1 ------------------------------------------------------------ 0

NC_045512.2 AGTCATTTTGCTATTGGCCTAGCTCTCTACTACCCTTCTGCTCGCATAGTGTATACAGCT 17160

MT186676.1 ------------------------------------------------------------ 0

NC_045512.2 TGCTCTCATGCCGCTGTTGATGCACTATGTGAGAAGGCATTAAAATATTTGCCTATAGAT 17220

MT186676.1 ------------------------------------------------------------ 0

NC_045512.2 AAATGTAGTAGAATTATACCTGCACGTGCTCGTGTAGAGTGTTTTGATAAATTCAAAGTG 17280

MT186676.1 ------------------------------------------------------------ 0

NC_045512.2 AATTCAACATTAGAACAGTATGTCTTTTGTACTGTAAATGCATTGCCTGAGACGACAGCA 17340

MT186676.1 ------------------------------------------------------------ 0

NC_045512.2 GATATAGTTGTCTTTGATGAAATTTCAATGGCCACAAATTATGATTTGAGTGTTGTCAAT 17400

MT186676.1 ------------------------------------------------------------ 0

NC_045512.2 GCCAGATTACGTGCTAAGCACTATGTGTACATTGGCGACCCTGCTCAATTACCTGCACCA 17460

MT186676.1 ------------------------------------------------------------ 0

NC_045512.2 CGCACATTGCTAACTAAGGGCACACTAGAACCAGAATATTTCAATTCAGTGTGTAGACTT 17520

MT186676.1 ------------------------------------------------------------ 0

NC_045512.2 ATGAAAACTATAGGTCCAGACATGTTCCTCGGAACTTGTCGGCGTTGTCCTGCTGAAATT 17580

MT186676.1 ------------------------------------------------------------ 0

NC_045512.2 GTTGACACTGTGAGTGCTTTGGTTTATGATAATAAGCTTAAAGCACATAAAGACAAATCA 17640

MT186676.1 ------------------------------------------------------------ 0

NC_045512.2 GCTCAATGCTTTAAAATGTTTTATAAGGGTGTTATCACGCATGATGTTTCATCTGCAATT 17700

MT186676.1 ------------------------------------------------------------ 0

NC_045512.2 AACAGGCCACAAATAGGCGTGGTAAGAGAATTCCTTACACGTAACCCTGCTTGGAGAAAA 17760

MT186676.1 ------------------------------------------------------------ 0

NC_045512.2 GCTGTCTTTATTTCACCTTATAATTCACAGAATGCTGTAGCCTCAAAGATTTTGGGACTA 17820

MT186676.1 ------------------------------------------------------------ 0

NC_045512.2 CCAACTCAAACTGTTGATTCATCACAGGGCTCAGAATATGACTATGTCATATTCACTCAA 17880

MT186676.1 ------------------------------------------------------------ 0

NC_045512.2 ACCACTGAAACAGCTCACTCTTGTAATGTAAACAGATTTAATGTTGCTATTACCAGAGCA 17940

MT186676.1 ------------------------------------------------------------ 0

NC_045512.2 AAAGTAGGCATACTTTGCATAATGTCTGATAGAGACCTTTATGACAAGTTGCAATTTACA 18000

MT186676.1 ------------------------------------------------------------ 0

NC_045512.2 AGTCTTGAAATTCCACGTAGGAATGTGGCAACTTTACAAGCTGAAAATGTAACAGGACTC 18060

MT186676.1 ------------------------------------------------------------ 0

NC_045512.2 TTTAAAGATTGTAGTAAGGTAATCACTGGGTTACATCCTACACAGGCACCTACACACCTC 18120

MT186676.1 ------------------------------------------------------------ 0

NC_045512.2 AGTGTTGACACTAAATTCAAAACTGAAGGTTTATGTGTTGACATACCTGGCATACCTAAG 18180

MT186676.1 ------------------------------------------------------------ 0

NC_045512.2 GACATGACCTATAGAAGACTCATCTCTATGATGGGTTTTAAAATGAATTATCAAGTTAAT 18240

MT186676.1 ------------------------------------------------------------ 0

NC_045512.2 GGTTACCCTAACATGTTTATCACCCGCGAAGAAGCTATAAGACATGTACGTGCATGGATT 18300

MT186676.1 ------------------------------------------------------------ 0

NC_045512.2 GGCTTCGATGTCGAGGGGTGTCATGCTACTAGAGAAGCTGTTGGTACCAATTTACCTTTA 18360

MT186676.1 ------------------------------------------------------------ 0

NC_045512.2 CAGCTAGGTTTTTCTACAGGTGTTAACCTAGTTGCTGTACCTACAGGTTATGTTGATACA 18420

MT186676.1 ------------------------------------------------------------ 0

NC_045512.2 CCTAATAATACAGATTTTTCCAGAGTTAGTGCTAAACCACCGCCTGGAGATCAATTTAAA 18480

MT186676.1 ------------------------------------------------------------ 0

NC_045512.2 CACCTCATACCACTTATGTACAAAGGACTTCCTTGGAATGTAGTGCGTATAAAGATTGTA 18540

MT186676.1 ------------------------------------------------------------ 0

NC_045512.2 CAAATGTTAAGTGACACACTTAAAAATCTCTCTGACAGAGTCGTATTTGTCTTATGGGCA 18600

MT186676.1 ------------------------------------------------------------ 0

NC_045512.2 CATGGCTTTGAGTTGACATCTATGAAGTATTTTGTGAAAATAGGACCTGAGCGCACCTGT 18660

MT186676.1 ------------------------------------------------------------ 0

NC_045512.2 TGTCTATGTGATAGACGTGCCACATGCTTTTCCACTGCTTCAGACACTTATGCCTGTTGG 18720

MT186676.1 ------------------------------------------------------------ 0

NC_045512.2 CATCATTCTATTGGATTTGATTACGTCTATAATCCGTTTATGATTGATGTTCAACAATGG 18780

MT186676.1 ------------------------------------------------------------ 0

NC_045512.2 GGTTTTACAGGTAACCTACAAAGCAACCATGATCTGTATTGTCAAGTCCATGGTAATGCA 18840

MT186676.1 ------------------------------------------------------------ 0

NC_045512.2 CATGTAGCTAGTTGTGATGCAATCATGACTAGGTGTCTAGCTGTCCACGAGTGCTTTGTT 18900

MT186676.1 ------------------------------------------------------------ 0

NC_045512.2 AAGCGTGTTGACTGGACTATTGAATATCCTATAATTGGTGATGAACTGAAGATTAATGCG 18960

MT186676.1 ------------------------------------------------------------ 0

NC_045512.2 GCTTGTAGAAAGGTTCAACACATGGTTGTTAAAGCTGCATTATTAGCAGACAAATTCCCA 19020

MT186676.1 ------------------------------------------------------------ 0

NC_045512.2 GTTCTTCACGACATTGGTAACCCTAAAGCTATTAAGTGTGTACCTCAAGCTGATGTAGAA 19080

MT186676.1 ------------------------------------------------------------ 0

NC_045512.2 TGGAAGTTCTATGATGCACAGCCTTGTAGTGACAAAGCTTATAAAATAGAAGAATTATTC 19140

MT186676.1 ------------------------------------------------------------ 0

NC_045512.2 TATTCTTATGCCACACATTCTGACAAATTCACAGATGGTGTATGCCTATTTTGGAATTGC 19200

MT186676.1 ------------------------------------------------------------ 0

NC_045512.2 AATGTCGATAGATATCCTGCTAATTCCATTGTTTGTAGATTTGACACTAGAGTGCTATCT 19260

MT186676.1 ------------------------------------------------------------ 0

NC_045512.2 AACCTTAACTTGCCTGGTTGTGATGGTGGCAGTTTGTATGTAAATAAACATGCATTCCAC 19320

MT186676.1 ------------------------------------------------------------ 0

NC_045512.2 ACACCAGCTTTTGATAAAAGTGCTTTTGTTAATTTAAAACAATTACCATTTTTCTATTAC 19380

MT186676.1 ------------------------------------------------------------ 0

NC_045512.2 TCTGACAGTCCATGTGAGTCTCATGGAAAACAAGTAGTGTCAGATATAGATTATGTACCA 19440

MT186676.1 ------------------------------------------------------------ 0

NC_045512.2 CTAAAGTCTGCTACGTGTATAACACGTTGCAATTTAGGTGGTGCTGTCTGTAGACATCAT 19500

MT186676.1 ------------------------------------------------------------ 0

NC_045512.2 GCTAATGAGTACAGATTGTATCTCGATGCTTATAACATGATGATCTCAGCTGGCTTTAGC 19560

MT186676.1 ------------------------------------------------------------ 0

NC_045512.2 TTGTGGGTTTACAAACAATTTGATACTTATAACCTCTGGAACACTTTTACAAGACTTCAG 19620

MT186676.1 ------------------------------------------------------------ 0

NC_045512.2 AGTTTAGAAAATGTGGCTTTTAATGTTGTAAATAAGGGACACTTTGATGGACAACAGGGT 19680

MT186676.1 ------------------------------------------------------------ 0

NC_045512.2 GAAGTACCAGTTTCTATCATTAATAACACTGTTTACACAAAAGTTGATGGTGTTGATGTA 19740

MT186676.1 ------------------------------------------------------------ 0

NC_045512.2 GAATTGTTTGAAAATAAAACAACATTACCTGTTAATGTAGCATTTGAGCTTTGGGCTAAG 19800

MT186676.1 ------------------------------------------------------------ 0

NC_045512.2 CGCAACATTAAACCAGTACCAGAGGTGAAAATACTCAATAATTTGGGTGTGGACATTGCT 19860

MT186676.1 ------------------------------------------------------------ 0

NC_045512.2 GCTAATACTGTGATCTGGGACTACAAAAGAGATGCTCCAGCACATATATCTACTATTGGT 19920

MT186676.1 ------------------------------------------------------------ 0

NC_045512.2 GTTTGTTCTATGACTGACATAGCCAAGAAACCAACTGAAACGATTTGTGCACCACTCACT 19980

MT186676.1 ------------------------------------------------------------ 0

NC_045512.2 GTCTTTTTTGATGGTAGAGTTGATGGTCAAGTAGACTTATTTAGAAATGCCCGTAATGGT 20040

MT186676.1 ------------------------------------------------------------ 0

NC_045512.2 GTTCTTATTACAGAAGGTAGTGTTAAAGGTTTACAACCATCTGTAGGTCCCAAACAAGCT 20100

MT186676.1 ------------------------------------------------------------ 0

NC_045512.2 AGTCTTAATGGAGTCACATTAATTGGAGAAGCCGTAAAAACACAGTTCAATTATTATAAG 20160

MT186676.1 ------------------------------------------------------------ 0

NC_045512.2 AAAGTTGATGGTGTTGTCCAACAATTACCTGAAACTTACTTTACTCAGAGTAGAAATTTA 20220

MT186676.1 ------------------------------------------------------------ 0

NC_045512.2 CAAGAATTTAAACCCAGGAGTCAAATGGAAATTGATTTCTTAGAATTAGCTATGGATGAA 20280

MT186676.1 ------------------------------------------------------------ 0

NC_045512.2 TTCATTGAACGGTATAAATTAGAAGGCTATGCCTTCGAACATATCGTTTATGGAGATTTT 20340

MT186676.1 ------------------------------------------------------------ 0

NC_045512.2 AGTCATAGTCAGTTAGGTGGTTTACATCTACTGATTGGACTAGCTAAACGTTTTAAGGAA 20400

MT186676.1 ------------------------------------------------------------ 0

NC_045512.2 TCACCTTTTGAATTAGAAGATTTTATTCCTATGGACAGTACAGTTAAAAACTATTTCATA 20460

MT186676.1 ------------------------------------------------------------ 0

NC_045512.2 ACAGATGCGCAAACAGGTTCATCTAAGTGTGTGTGTTCTGTTATTGATTTATTACTTGAT 20520

MT186676.1 ------------------------------------------------------------ 0

NC_045512.2 GATTTTGTTGAAATAATAAAATCCCAAGATTTATCTGTAGTTTCTAAGGTTGTCAAAGTG 20580

MT186676.1 ------------------------------------------------------------ 0

NC_045512.2 ACTATTGACTATACAGAAATTTCATTTATGCTTTGGTGTAAAGATGGCCATGTAGAAACA 20640

MT186676.1 ------------------------------------------------------------ 0

NC_045512.2 TTTTACCCAAAATTACAATCTAGTCAAGCGTGGCAACCGGGTGTTGCTATGCCTAATCTT 20700

MT186676.1 ------------------------------------------------------------ 0

NC_045512.2 TACAAAATGCAAAGAATGCTATTAGAAAAGTGTGACCTTCAAAATTATGGTGATAGTGCA 20760

MT186676.1 ------------------------------------------------------------ 0

NC_045512.2 ACATTACCTAAAGGCATAATGATGAATGTCGCAAAATATACTCAACTGTGTCAATATTTA 20820

MT186676.1 ------------------------------------------------------------ 0

NC_045512.2 AACACATTAACATTAGCTGTACCCTATAATATGAGAGTTATACATTTTGGTGCTGGTTCT 20880

MT186676.1 ------------------------------------------------------------ 0

NC_045512.2 GATAAAGGAGTTGCACCAGGTACAGCTGTTTTAAGACAGTGGTTGCCTACGGGTACGCTG 20940

MT186676.1 ------------------------------------------------------------ 0

NC_045512.2 CTTGTCGATTCAGATCTTAATGACTTTGTCTCTGATGCAGATTCAACTTTGATTGGTGAT 21000

MT186676.1 ------------------------------------------------------------ 0

NC_045512.2 TGTGCAACTGTACATACAGCTAATAAATGGGATCTCATTATTAGTGATATGTACGACCCT 21060

MT186676.1 ------------------------------------------------------------ 0

NC_045512.2 AAGACTAAAAATGTTACAAAAGAAAATGACTCTAAAGAGGGTTTTTTCACTTACATTTGT 21120

MT186676.1 ------------------------------------------------------------ 0

NC_045512.2 GGGTTTATACAACAAAAGCTAGCTCTTGGAGGTTCCGTGGCTATAAAGATAACAGAACAT 21180

MT186676.1 ------------------------------------------------------------ 0

NC_045512.2 TCTTGGAATGCTGATCTTTATAAGCTCATGGGACACTTCGCATGGTGGACAGCCTTTGTT 21240

MT186676.1 ------------------------------------------------------------ 0

NC_045512.2 ACTAATGTGAATGCGTCATCATCTGAAGCATTTTTAATTGGATGTAATTATCTTGGCAAA 21300

MT186676.1 ------------------------------------------------------------ 0

NC_045512.2 CCACGCGAACAAATAGATGGTTATGTCATGCATGCAAATTACATATTTTGGAGGAATACA 21360

MT186676.1 ------------------------------------------------------------ 0

NC_045512.2 AATCCAATTCAGTTGTCTTCCTATTCTTTATTTGACATGAGTAAATTTCCCCTTAAATTA 21420

MT186676.1 ------------------------------------------------------------ 0

NC_045512.2 AGGGGTACTGCTGTTATGTCTTTAAAAGAAGGTCAAATCAATGATATGATTTTATCTCTT 21480

MT186676.1 ------------------------------------------------------------ 0

NC_045512.2 CTTAGTAAAGGTAGACTTATAATTAGAGAAAACAACAGAGTTGTTATTTCTAGTGATGTT 21540

MT186676.1 ------------------------------------------------------------ 0

NC_045512.2 CTTGTTAACAACTAAACGAACAATGTTTGTTTTTCTTGTTTTATTGCCACTAGTCTCTAG 21600

MT186676.1 ------------------------------------------------------------ 0

NC_045512.2 TCAGTGTGTTAATCTTACAACCAGAACTCAATTACCCCCTGCATACACTAATTCTTTCAC 21660

MT186676.1 ------------------------------------------------------------ 0

NC_045512.2 ACGTGGTGTTTATTACCCTGACAAAGTTTTCAGATCCTCAGTTTTACATTCAACTCAGGA 21720

MT186676.1 ------------------------------------------------------------ 0

NC_045512.2 CTTGTTCTTACCTTTCTTTTCCAATGTTACTTGGTTCCATGCTATACATGTCTCTGGGAC 21780

MT186676.1 ------------------------------------------------------------ 0

NC_045512.2 CAATGGTACTAAGAGGTTTGATAACCCTGTCCTACCATTTAATGATGGTGTTTATTTTGC 21840

MT186676.1 ------------------------------------------------------------ 0

NC_045512.2 TTCCACTGAGAAGTCTAACATAATAAGAGGCTGGATTTTTGGTACTACTTTAGATTCGAA 21900

MT186676.1 ------------------------------------------------------------ 0

NC_045512.2 GACCCAGTCCCTACTTATTGTTAATAACGCTACTAATGTTGTTATTAAAGTCTGTGAATT 21960

MT186676.1 ------------------------------------------------------------ 0

NC_045512.2 TCAATTTTGTAATGATCCATTTTTGGGTGTTTATTACCACAAAAACAACAAAAGTTGGAT 22020

MT186676.1 ------------------------------------------------------------ 0

NC_045512.2 GGAAAGTGAGTTCAGAGTTTATTCTAGTGCGAATAATTGCACTTTTGAATATGTCTCTCA 22080

MT186676.1 ------------------------------------------------------------ 0

NC_045512.2 GCCTTTTCTTATGGACCTTGAAGGAAAACAGGGTAATTTCAAAAATCTTAGGGAATTTGT 22140

MT186676.1 ------------------------------------------------------------ 0

NC_045512.2 GTTTAAGAATATTGATGGTTATTTTAAAATATATTCTAAGCACACGCCTATTAATTTAGT 22200

MT186676.1 ------------------------------------------------------------ 0

NC_045512.2 GCGTGATCTCCCTCAGGGTTTTTCGGCTTTAGAACCATTGGTAGATTTGCCAATAGGTAT 22260

MT186676.1 ------------------------------------------------------------ 0

NC_045512.2 TAACATCACTAGGTTTCAAACTTTACTTGCTTTACATAGAAGTTATTTGACTCCTGGTGA 22320

MT186676.1 ------------------------------------------------------------ 0

NC_045512.2 TTCTTCTTCAGGTTGGACAGCTGGTGCTGCAGCTTATTATGTGGGTTATCTTCAACCTAG 22380

MT186676.1 ------------------------------------------------------------ 0

NC_045512.2 GACTTTTCTATTAAAATATAATGAAAATGGAACCATTACAGATGCTGTAGACTGTGCACT 22440

MT186676.1 ------------------------------------------------------------ 0

NC_045512.2 TGACCCTCTCTCAGAAACAAAGTGTACGTTGAAATCCTTCACTGTAGAAAAAGGAATCTA 22500

MT186676.1 ------------------------------------------------------------ 0

NC_045512.2 TCAAACTTCTAACTTTAGAGTCCAACCAACAGAATCTATTGTTAGATTTCCTAATATTAC 22560

MT186676.1 ------------------------------------------------------------ 0

NC_045512.2 AAACTTGTGCCCTTTTGGTGAAGTTTTTAACGCCACCAGATTTGCATCTGTTTATGCTTG 22620

MT186676.1 ------------------------------------------------------------ 0

NC_045512.2 GAACAGGAAGAGAATCAGCAACTGTGTTGCTGATTATTCTGTCCTATATAATTCCGCATC 22680

MT186676.1 ------------------------------------------------------------ 0

NC_045512.2 ATTTTCCACTTTTAAGTGTTATGGAGTGTCTCCTACTAAATTAAATGATCTCTGCTTTAC 22740

MT186676.1 ------------------------------------------------------------ 0

NC_045512.2 TAATGTCTATGCAGATTCATTTGTAATTAGAGGTGATGAAGTCAGACAAATCGCTCCAGG 22800

MT186676.1 ------------------------------------------------------------ 0

NC_045512.2 GCAAACTGGAAAGATTGCTGATTATAATTATAAATTACCAGATGATTTTACAGGCTGCGT 22860

MT186676.1 ------------------------------------------------------------ 0

NC_045512.2 TATAGCTTGGAATTCTAACAATCTTGATTCTAAGGTTGGTGGTAATTATAATTACCTGTA 22920

MT186676.1 ------------------------------------------------------------ 0

NC_045512.2 TAGATTGTTTAGGAAGTCTAATCTCAAACCTTTTGAGAGAGATATTTCAACTGAAATCTA 22980

MT186676.1 ------------------------------------------------------------ 0

NC_045512.2 TCAGGCCGGTAGCACACCTTGTAATGGTGTTGAAGGTTTTAATTGTTACTTTCCTTTACA 23040

MT186676.1 ------------------------------------------------------------ 0

NC_045512.2 ATCATATGGTTTCCAACCCACTAATGGTGTTGGTTACCAACCATACAGAGTAGTAGTACT 23100

MT186676.1 ------------------------------------------------------------ 0

NC_045512.2 TTCTTTTGAACTTCTACATGCACCAGCAACTGTTTGTGGACCTAAAAAGTCTACTAATTT 23160

MT186676.1 ------------------------------------------------------------ 0

NC_045512.2 GGTTAAAAACAAATGTGTCAATTTCAACTTCAATGGTTTAACAGGCACAGGTGTTCTTAC 23220

MT186676.1 ------------------------------------------------------------ 0

NC_045512.2 TGAGTCTAACAAAAAGTTTCTGCCTTTCCAACAATTTGGCAGAGACATTGCTGACACTAC 23280

MT186676.1 ------------------------------------------------------------ 0

NC_045512.2 TGATGCTGTCCGTGATCCACAGACACTTGAGATTCTTGACATTACACCATGTTCTTTTGG 23340

MT186676.1 ------------------------------------------------------------ 0

NC_045512.2 TGGTGTCAGTGTTATAACACCAGGAACAAATACTTCTAACCAGGTTGCTGTTCTTTATCA 23400

MT186676.1 ------------------------------------------------------------ 0

NC_045512.2 GGATGTTAACTGCACAGAAGTCCCTGTTGCTATTCATGCAGATCAACTTACTCCTACTTG 23460

MT186676.1 ------------------------------------------------------------ 0

NC_045512.2 GCGTGTTTATTCTACAGGTTCTAATGTTTTTCAAACACGTGCAGGCTGTTTAATAGGGGC 23520

MT186676.1 ------------------------------------------------------------ 0

NC_045512.2 TGAACATGTCAACAACTCATATGAGTGTGACATACCCATTGGTGCAGGTATATGCGCTAG 23580

MT186676.1 ------------------------------------------------------------ 0

NC_045512.2 TTATCAGACTCAGACTAATTCTCCTCGGCGGGCACGTAGTGTAGCTAGTCAATCCATCAT 23640

MT186676.1 ------------------------------------------------------------ 0

NC_045512.2 TGCCTACACTATGTCACTTGGTGCAGAAAATTCAGTTGCTTACTCTAATAACTCTATTGC 23700

MT186676.1 ------------------------------------------------------------ 0

NC_045512.2 CATACCCACAAATTTTACTATTAGTGTTACCACAGAAATTCTACCAGTGTCTATGACCAA 23760

MT186676.1 ------------------------------------------------------------ 0

NC_045512.2 GACATCAGTAGATTGTACAATGTACATTTGTGGTGATTCAACTGAATGCAGCAATCTTTT 23820

MT186676.1 ------------------------------------------------------------ 0

NC_045512.2 GTTGCAATATGGCAGTTTTTGTACACAATTAAACCGTGCTTTAACTGGAATAGCTGTTGA 23880

MT186676.1 ------------------------------------------------------------ 0

NC_045512.2 ACAAGACAAAAACACCCAAGAAGTTTTTGCACAAGTCAAACAAATTTACAAAACACCACC 23940

MT186676.1 ------------------------------------------------------------ 0

NC_045512.2 AATTAAAGATTTTGGTGGTTTTAATTTTTCACAAATATTACCAGATCCATCAAAACCAAG 24000

MT186676.1 ------------------------------------------------------------ 0

NC_045512.2 CAAGAGGTCATTTATTGAAGATCTACTTTTCAACAAAGTGACACTTGCAGATGCTGGCTT 24060

MT186676.1 ------------------------------------------------------------ 0

NC_045512.2 CATCAAACAATATGGTGATTGCCTTGGTGATATTGCTGCTAGAGACCTCATTTGTGCACA 24120

MT186676.1 ------------------------------------------------------------ 0

NC_045512.2 AAAGTTTAACGGCCTTACTGTTTTGCCACCTTTGCTCACAGATGAAATGATTGCTCAATA 24180

MT186676.1 ------------------------------------------------------------ 0

NC_045512.2 CACTTCTGCACTGTTAGCGGGTACAATCACTTCTGGTTGGACCTTTGGTGCAGGTGCTGC 24240

MT186676.1 ------------------------------------------------------------ 0

NC_045512.2 ATTACAAATACCATTTGCTATGCAAATGGCTTATAGGTTTAATGGTATTGGAGTTACACA 24300

MT186676.1 ------------------------------------------------------------ 0

NC_045512.2 GAATGTTCTCTATGAGAACCAAAAATTGATTGCCAACCAATTTAATAGTGCTATTGGCAA 24360

MT186676.1 ------------------------------------------------------------ 0

NC_045512.2 AATTCAAGACTCACTTTCTTCCACAGCAAGTGCACTTGGAAAACTTCAAGATGTGGTCAA 24420

MT186676.1 ------------------------------------------------------------ 0

NC_045512.2 CCAAAATGCACAAGCTTTAAACACGCTTGTTAAACAACTTAGCTCCAATTTTGGTGCAAT 24480

MT186676.1 ------------------------------------------------------------ 0

NC_045512.2 TTCAAGTGTTTTAAATGATATCCTTTCACGTCTTGACAAAGTTGAGGCTGAAGTGCAAAT 24540

MT186676.1 ------------------------------------------------------------ 0

NC_045512.2 TGATAGGTTGATCACAGGCAGACTTCAAAGTTTGCAGACATATGTGACTCAACAATTAAT 24600

MT186676.1 ------------------------------------------------------------ 0

NC_045512.2 TAGAGCTGCAGAAATCAGAGCTTCTGCTAATCTTGCTGCTACTAAAATGTCAGAGTGTGT 24660

MT186676.1 ------------------------------------------------------------ 0

NC_045512.2 ACTTGGACAATCAAAAAGAGTTGATTTTTGTGGAAAGGGCTATCATCTTATGTCCTTCCC 24720

MT186676.1 ------------------------------------------------------------ 0

NC_045512.2 TCAGTCAGCACCTCATGGTGTAGTCTTCTTGCATGTGACTTATGTCCCTGCACAAGAAAA 24780

MT186676.1 ------------------------------------------------------------ 0

NC_045512.2 GAACTTCACAACTGCTCCTGCCATTTGTCATGATGGAAAAGCACACTTTCCTCGTGAAGG 24840

MT186676.1 ------------------------------------------------------------ 0

NC_045512.2 TGTCTTTGTTTCAAATGGCACACACTGGTTTGTAACACAAAGGAATTTTTATGAACCACA 24900

MT186676.1 ------------------------------------------------------------ 0

NC_045512.2 AATCATTACTACAGACAACACATTTGTGTCTGGTAACTGTGATGTTGTAATAGGAATTGT 24960

MT186676.1 ------------------------------------------------------------ 0

NC_045512.2 CAACAACACAGTTTATGATCCTTTGCAACCTGAATTAGACTCATTCAAGGAGGAGTTAGA 25020

MT186676.1 ------------------------------------------------------------ 0

NC_045512.2 TAAATATTTTAAGAATCATACATCACCAGATGTTGATTTAGGTGACATCTCTGGCATTAA 25080

MT186676.1 ------------------------------------------------------------ 0

NC_045512.2 TGCTTCAGTTGTAAACATTCAAAAAGAAATTGACCGCCTCAATGAGGTTGCCAAGAATTT 25140

MT186676.1 ------------------------------------------------------------ 0

NC_045512.2 AAATGAATCTCTCATCGATCTCCAAGAACTTGGAAAGTATGAGCAGTATATAAAATGGCC 25200

MT186676.1 ------------------------------------------------------------ 0

NC_045512.2 ATGGTACATTTGGCTAGGTTTTATAGCTGGCTTGATTGCCATAGTAATGGTGACAATTAT 25260

MT186676.1 ------------------------------------------------------------ 0

NC_045512.2 GCTTTGCTGTATGACCAGTTGCTGTAGTTGTCTCAAGGGCTGTTGTTCTTGTGGATCCTG 25320

MT186676.1 ------------------------------------------------------------ 0

NC_045512.2 CTGCAAATTTGATGAAGACGACTCTGAGCCAGTGCTCAAAGGAGTCAAATTACATTACAC 25380

MT186676.1 ------------------------------------------------------------ 0

NC_045512.2 ATAAACGAACTTATGGATTTGTTTATGAGAATCTTCACAATTGGAACTGTAACTTTGAAG 25440

MT186676.1 ------------------------------------------------------------ 0

NC_045512.2 CAAGGTGAAATCAAGGATGCTACTCCTTCAGATTTTGTTCGCGCTACTGCAACGATACCG 25500

MT186676.1 ------------------------------------------------------------ 0

NC_045512.2 ATACAAGCCTCACTCCCTTTCGGATGGCTTATTGTTGGCGTTGCACTTCTTGCTGTTTTT 25560

MT186676.1 ------------------------------------------------------------ 0

NC_045512.2 CAGAGCGCTTCCAAAATCATAACCCTCAAAAAGAGATGGCAACTAGCACTCTCCAAGGGT 25620

MT186676.1 ------------------------------------------------------------ 0

NC_045512.2 GTTCACTTTGTTTGCAACTTGCTGTTGTTGTTTGTAACAGTTTACTCACACCTTTTGCTC 25680

MT186676.1 ------------------------------------------------------------ 0

NC_045512.2 GTTGCTGCTGGCCTTGAAGCCCCTTTTCTCTATCTTTATGCTTTAGTCTACTTCTTGCAG 25740

MT186676.1 ------------------------------------------------------------ 0

NC_045512.2 AGTATAAACTTTGTAAGAATAATAATGAGGCTTTGGCTTTGCTGGAAATGCCGTTCCAAA 25800

MT186676.1 ------------------------------------------------------------ 0

NC_045512.2 AACCCATTACTTTATGATGCCAACTATTTTCTTTGCTGGCATACTAATTGTTACGACTAT 25860

MT186676.1 ------------------------------------------------------------ 0

NC_045512.2 TGTATACCTTACAATAGTGTAACTTCTTCAATTGTCATTACTTCAGGTGATGGCACAACA 25920

MT186676.1 ------------------------------------------------------------ 0

NC_045512.2 AGTCCTATTTCTGAACATGACTACCAGATTGGTGGTTATACTGAAAAATGGGAATCTGGA 25980

MT186676.1 ------------------------------------------------------------ 0

NC_045512.2 GTAAAAGACTGTGTTGTATTACACAGTTACTTCACTTCAGACTATTACCAGCTGTACTCA 26040

MT186676.1 ------------------------------------------------------------ 0

NC_045512.2 ACTCAATTGAGTACAGACACTGGTGTTGAACATGTTACCTTCTTCATCTACAATAAAATT 26100

MT186676.1 ------------------------------------------------------------ 0

NC_045512.2 GTTGATGAGCCTGAAGAACATGTCCAAATTCACACAATCGACGGTTCATCCGGAGTTGTT 26160

MT186676.1 ------------------------------------------------------------ 0

NC_045512.2 AATCCAGTAATGGAACCAATTTATGATGAACCGACGACGACTACTAGCGTGCCTTTGTAA 26220

MT186676.1 ------------------------------------------------------------ 0

NC_045512.2 GCACAAGCTGATGAGTACGAACTTATGTACTCATTCGTTTCGGAAGAGACAGGTACGTTA 26280

MT186676.1 ------------------------------------------------------------ 0

NC_045512.2 ATAGTTAATAGCGTACTTCTTTTTCTTGCTTTCGTGGTATTCTTGCTAGTTACACTAGCC 26340

MT186676.1 ------------------------------------------------------------ 0

NC_045512.2 ATCCTTACTGCGCTTCGATTGTGTGCGTACTGCTGCAATATTGTTAACGTGAGTCTTGTA 26400

MT186676.1 ------------------------------------------------------------ 0

NC_045512.2 AAACCTTCTTTTTACGTTTACTCTCGTGTTAAAAATCTGAATTCTTCTAGAGTTCCTGAT 26460

MT186676.1 ------------------------------------------------------------ 0

NC_045512.2 CTTCTGGTCTAAACGAACTAAATATTATATTAGTTTTTCTGTTTGGAACTTTAATTTTAG 26520

MT186676.1 ------------------------------------------------------------ 0

NC_045512.2 CCATGGCAGATTCCAACGGTACTATTACCGTTGAAGAGCTTAAAAAGCTCCTTGAACAAT 26580

MT186676.1 ------------------------------------------------------------ 0

NC_045512.2 GGAACCTAGTAATAGGTTTCCTATTCCTTACATGGATTTGTCTTCTACAATTTGCCTATG 26640

MT186676.1 ------------------------------------------------------------ 0

NC_045512.2 CCAACAGGAATAGGTTTTTGTATATAATTAAGTTAATTTTCCTCTGGCTGTTATGGCCAG 26700

MT186676.1 ------------------------------------------------------------ 0

NC_045512.2 TAACTTTAGCTTGTTTTGTGCTTGCTGCTGTTTACAGAATAAATTGGATCACCGGTGGAA 26760

MT186676.1 ------------------------------------------------------------ 0

NC_045512.2 TTGCTATCGCAATGGCTTGTCTTGTAGGCTTGATGTGGCTCAGCTACTTCATTGCTTCTT 26820

MT186676.1 ------------------------------------------------------------ 0

NC_045512.2 TCAGACTGTTTGCGCGTACGCGTTCCATGTGGTCATTCAATCCAGAAACTAACATTCTTC 26880

MT186676.1 ------------------------------------------------------------ 0

NC_045512.2 TCAACGTGCCACTCCATGGCACTATTCTGACCAGACCGCTTCTAGAAAGTGAACTCGTAA 26940

MT186676.1 ------------------------------------------------------------ 0

NC_045512.2 TCGGAGCTGTGATCCTTCGTGGACATCTTCGTATTGCTGGACACCATCTAGGACGCTGTG 27000

MT186676.1 ------------------------------------------------------------ 0

NC_045512.2 ACATCAAGGACCTGCCTAAAGAAATCACTGTTGCTACATCACGAACGCTTTCTTATTACA 27060

MT186676.1 ------------------------------------------------------------ 0

NC_045512.2 AATTGGGAGCTTCGCAGCGTGTAGCAGGTGACTCAGGTTTTGCTGCATACAGTCGCTACA 27120

MT186676.1 ------------------------------------------------------------ 0

NC_045512.2 GGATTGGCAACTATAAATTAAACACAGACCATTCCAGTAGCAGTGACAATATTGCTTTGC 27180

MT186676.1 ------------------------------------------------------------ 0

NC_045512.2 TTGTACAGTAAGTGACAACAGATGTTTCATCTCGTTGACTTTCAGGTTACTATAGCAGAG 27240

MT186676.1 ------------------------------------------------------------ 0

NC_045512.2 ATATTACTAATTATTATGAGGACTTTTAAAGTTTCCATTTGGAATCTTGATTACATCATA 27300

MT186676.1 ------------------------------------------------------------ 0

NC_045512.2 AACCTCATAATTAAAAATTTATCTAAGTCACTAACTGAGAATAAATATTCTCAATTAGAT 27360

MT186676.1 ------------------------------------------------------------ 0

NC_045512.2 GAAGAGCAACCAATGGAGATTGATTAAACGAACATGAAAATTATTCTTTTCTTGGCACTG 27420

MT186676.1 ------------------------------------------------------------ 0

NC_045512.2 ATAACACTCGCTACTTGTGAGCTTTATCACTACCAAGAGTGTGTTAGAGGTACAACAGTA 27480

MT186676.1 ------------------------------------------------------------ 0

NC_045512.2 CTTTTAAAAGAACCTTGCTCTTCTGGAACATACGAGGGCAATTCACCATTTCATCCTCTA 27540

MT186676.1 ------------------------------------------------------------ 0

NC_045512.2 GCTGATAACAAATTTGCACTGACTTGCTTTAGCACTCAATTTGCTTTTGCTTGTCCTGAC 27600

MT186676.1 ------------------------------------------------------------ 0

NC_045512.2 GGCGTAAAACACGTCTATCAGTTACGTGCCAGATCAGTTTCACCTAAACTGTTCATCAGA 27660

MT186676.1 ------------------------------------------------------------ 0

NC_045512.2 CAAGAGGAAGTTCAAGAACTTTACTCTCCAATTTTTCTTATTGTTGCGGCAATAGTGTTT 27720

MT186676.1 ------------------------------------------------------------ 0

NC_045512.2 ATAACACTTTGCTTCACACTCAAAAGAAAGACAGAATGATTGAACTTTCATTAATTGACT 27780

MT186676.1 ------------------------------------------------------------ 0

NC_045512.2 TCTATTTGTGCTTTTTAGCCTTTCTGCTATTCCTTGTTTTAATTATGCTTATTATCTTTT 27840

MT186676.1 ------------------------------------------------------------ 0

NC_045512.2 GGTTCTCACTTGAACTGCAAGATCATAATGAAACTTGTCACGCCTAAACGAACATGAAAT 27900

MT186676.1 ------------------------------------------------------------ 0

NC_045512.2 TTCTTGTTTTCTTAGGAATCATCACAACTGTAGCTGCATTTCACCAAGAATGTAGTTTAC 27960

MT186676.1 ------------------------------------------------------------ 0

NC_045512.2 AGTCATGTACTCAACATCAACCATATGTAGTTGATGACCCGTGTCCTATTCACTTCTATT 28020

MT186676.1 ------------------------------------------------------------ 0

NC_045512.2 CTAAATGGTATATTAGAGTAGGAGCTAGAAAATCAGCACCTTTAATTGAATTGTGCGTGG 28080

MT186676.1 ------------------------------------------------------------ 0

NC_045512.2 ATGAGGCTGGTTCTAAATCACCCATTCAGTACATCGATATCGGTAATTATACAGTTTCCT 28140

MT186676.1 ------------------------------------------------------------ 0

NC_045512.2 GTTTACCTTTTACAATTAATTGCCAGGAACCTAAATTGGGTAGTCTTGTAGTGCGTTGTT 28200

MT186676.1 ------------------------------------------------------------ 0

NC_045512.2 CGTTCTATGAAGACTTTTTAGAGTATCATGACGTTCGTGTTGTTTTAGATTTCATCTAAA 28260

MT186676.1 ------------------------------------------------------------ 0

NC_045512.2 CGAACAAACTAAAATGTCTGATAATGGACCCCAAAATCAGCGAAATGCACCCCGCATTAC 28320

MT186676.1 ------------------------------------------------------------ 0

NC_045512.2 GTTTGGTGGACCCTCAGATTCAACTGGCAGTAACCAGAATGGAGAACGCAGTGGGGCGCG 28380

MT186676.1 --------------------------------------------------------CGCG 4

****

NC_045512.2 ATCAAAACAACGTCGGCCCCAAGGTTTACCCAATAATACTGCGTCTTGGTTCACCGCTCT 28440

MT186676.1 ATCAAAACAACGTCGGCCCCAAGGTTTACCCAATAATACTGCGTCTTGGTTCACCGCTCT 64

************************************************************

NC_045512.2 CACTCAACATGGCAAGGAAGACCTTAAATTCCCTCGAGGACAAGGCGTTCCAATTAACAC 28500

MT186676.1 CACTCAACATGGCAAGGAAGACCTTAAATTCCCTCGAGGACAAGGCGTTCCAATTAACAC 124

************************************************************

NC_045512.2 CAATAGCAGTCCAGATGACCAAATTGGCTACTACCGAAGAGCTACCAGACGAATTCGTGG 28560

MT186676.1 CAATAGCAGTCCAGATGACCAAATTGGCTACTACCGAAGAGCTACCAGACGAATTCGTGG 184

************************************************************

NC_045512.2 TGGTGACGGTAAAATGAAAGATCTCAGTCCAAGATGGTATTTCTACTACCTAGGAACTGG 28620

MT186676.1 TGGTGACGGTAAAATGAAAGATCTCAGTCCAAGATGGTATTTCTACTACCTAGGAACTGG 244

************************************************************

NC_045512.2 GCCAGAAGCTGGACTTCCCTATGGTGCTAACAAAGACGGCATCATATGGGTTGCAACTGA 28680

MT186676.1 GCCAGAAGCTGGACTTCCCTATGGTGCTAACAAAGACGGCATCATATGGGTTGCAACTGA 304

************************************************************

NC_045512.2 GGGAGCC**T**TGAATACACCAAAAGATCACATTGGCACCCGCAATCCTGCTAACAATGCTGC 28740

MT186676.1 GGGAGCC**C**TGAATACACCAAAAGATCACATTGGCACCCGCAATCCTGCTAACAATGCTG- 363

******* ***************************************************

NC_045512.2 AATCGTGCTACAACTTCCTCAAGGAACAACATTGCCAAAAGGCTTCTACGCAGAAGGGAG 28800

MT186676.1 ------------------------------------------------------------ 363

NC_045512.2 CAGAGGCGGCAGTCAAGCCTCTTCTCGTTCCTCATCACGTAGTCGCAACAGTTCAAGAAA 28860

MT186676.1 ------------------------------------------------------------ 363

NC_045512.2 TTCAACTCCAGGCAGCAGTAGGGGAACTTCTCCTGCTAGAATGGCTGGCAATGGCGGTGA 28920

MT186676.1 ------------------------------------------------------------ 363

NC_045512.2 TGCTGCTCTTGCTTTGCTGCTGCTTGACAGATTGAACCAGCTTGAGAGCAAAATGTCTGG 28980

MT186676.1 ------------------------------------------------------------ 363

NC_045512.2 TAAAGGCCAACAACAACAAGGCCAAACTGTCACTAAGAAATCTGCTGCTGAGGCTTCTAA 29040

MT186676.1 ------------------------------------------------------------ 363

NC_045512.2 GAAGCCTCGGCAAAAACGTACTGCCACTAAAGCATACAATGTAACACAAGCTTTCGGCAG 29100

MT186676.1 ------------------------------------------------------------ 363

NC_045512.2 ACGTGGTCCAGAACAAACCCAAGGAAATTTTGGGGACCAGGAACTAATCAGACAAGGAAC 29160

MT186676.1 ------------------------------------------------------------ 363

NC_045512.2 TGATTACAAACATTGGCCGCAAATTGCACAATTTGCCCCCAGCGCTTCAGCGTTCTTCGG 29220

MT186676.1 ------------------------------------------------------------ 363

NC_045512.2 AATGTCGCGCATTGGCATGGAAGTCACACCTTCGGGAACGTGGTTGACCTACACAGGTGC 29280

MT186676.1 ------------------------------------------------------------ 363

NC_045512.2 CATCAAATTGGATGACAAAGATCCAAATTTCAAAGATCAAGTCATTTTGCTGAATAAGCA 29340

MT186676.1 ------------------------------------------------------------ 363

NC_045512.2 TATTGACGCATACAAAACATTCCCACCAACAGAGCCTAAAAAGGACAAAAAGAAGAAGGC 29400

MT186676.1 ------------------------------------------------------------ 363

NC_045512.2 TGATGAAACTCAAGCCTTACCGCAGAGACAGAAGAAACAGCAAACTGTGACTCTTCTTCC 29460

MT186676.1 ------------------------------------------------------------ 363

NC_045512.2 TGCTGCAGATTTGGATGATTTCTCCAAACAATTGCAACAATCCATGAGCAGTGCTGACTC 29520

MT186676.1 ------------------------------------------------------------ 363

NC_045512.2 AACTCAGGCCTAAACTCATGCAGACCACACAAGGCAGATGGGCTATATAAACGTTTTCGC 29580

MT186676.1 ------------------------------------------------------------ 363

NC_045512.2 TTTTCCGTTTACGATATATAGTCTACTCTTGTGCAGAATGAATTCTCGTAACTACATAGC 29640

MT186676.1 ------------------------------------------------------------ 363

NC_045512.2 ACAAGTAGATGTAGTTAACTTTAATCTCACATAGCAATCTTTAATCAGTGTGTAACATTA 29700

MT186676.1 ------------------------------------------------------------ 363

NC_045512.2 GGGAGGACTTGAAAGAGCCACCACATTTTCACCGAGGCCACGCGGAGTACGATCGAGTGT 29760

MT186676.1 ------------------------------------------------------------ 363

NC_045512.2 ACAGTGAACAATGCTAGGGAGAGCTGCCTATATGGAAGAGCCCTAATGTGTAAAATTAAT 29820

MT186676.1 ------------------------------------------------------------ 363

NC_045512.2 TTTAGTAGTGCTATCCCCATGTGATTTTAATAGCTTCTTAGGAGAATGACAAAAAAAAAA 29880

MT186676.1 ------------------------------------------------------------ 363

NC_045512.2 AAAAAAAAAAAAAAAAAAAAAAA 29903

MT186676.1 ----------------------- 363

**(B)** **MSA between the protein sequence of the RNA binding domain of N protein from Wuhan, China, and the protein sequence of the RNA binding domain of N protein from Iran.**

QIE07458.1 MSDNGPQNQRNAPRITFGGPSDSTGSNQNGERSGARSKQRRPQGLPNNTASWFTALTQHG 60

QIK02784.1 -----------------------------------RSKQRRPQGLPNNTASWFTALTQHG 25

*************************

QIE07458.1 KEDLKFPRGQGVPINTNSSPDDQIGYYRRATRRIRGGDGKMKDLSPRWYFYYLGTGPEAG 120

QIK02784.1 KEDLKFPRGQGVPINTNSSPDDQIGYYRRATRRIRGGDGKMKDLSPRWYFYYLGTGPEAG 85

************************************************************

QIE07458.1 LPYGANKDGIIWVATEGALNTPKDHIGTRNPANNAAIVLQLPQGTTLPKGFYAEGSRGGS 180

QIK02784.1 LPYGANKDGIIWVATEGALNTPKDHIGTRNPANNA------------------------- 120

***********************************

QIE07458.1 QASSRSSSRSRNSSRNSTPGSSRGTSPARMAGNGGDAALALLLLDRLNQLESKMSGKGQQ 240

QIK02784.1 ------------------------------------------------------------ 120

QIE07458.1 QQGQTVTKKSAAEASKKPRQKRTATKAYNVTQAFGRRGPEQTQGNFGDQELIRQGTDYKH 300

QIK02784.1 ------------------------------------------------------------ 120

QIE07458.1 WPQIAQFAPSASAFFGMSRIGMEVTPSGTWLTYTGAIKLDDKDSNFKDQVILLNKHIDAY 360

QIK02784.1 ------------------------------------------------------------ 120

QIE07458.1 KTFPPTEPKKDKKKKADETQALPQRQKKQQTVTLLPAADLDDFSKQLQQSMSSADSTQA 419

QIK02784.1 ----------------------------------------------------------- 120
